# Description of the immature stages of *Larinus vulpes* and notes on its biology (Coleoptera, Curculionidae, Lixinae)

**DOI:** 10.3897/zookeys.679.12560

**Published:** 2017-06-12

**Authors:** Jiří Skuhrovec, Semyon Volovnik, Rafał Gosik

**Affiliations:** 1 Group Function of Invertebrate and Plant Biodiversity in Agro-Ecosystems, Crop Research Institute, Drnovská 507, CZ-161 06 Praha 6 – Ruzyně, Czech Republic; 2 Independent Researcher, Melitopol, Ukraine; 3 Department of Zoology, Maria Curie-Skłodowska University, Akademicka 19, 20-033 Lublin, Poland

**Keywords:** Asteraceae, *Echinops*, eastern Europe, host plant, larva, larval development, life history, morphology, Palaearctic Region, pupa, weevil

## Abstract

Mature larva and pupa of *Larinus vulpes* (Olivier, 1807) (Curculionidae: Lixinae: Lixini) are morphologically described for the first time and compared with known larvae and pupae of other *Larinus* species. Very high counts of larval body setae (pronotum with more than 25 setae and postdorsum on meso- and metathorax and also on abdominal segments I–VII with more than 12 setae) are characteristic features of the nominotypical subgenus Larinus. The biology of the species was studied in Ukraine. *Echinops ruthenicus* and *E. sphaerocephalus* were identified as host plants of both larvae and adults of this weevil based on the present research in Ukraine, which shows probably oligophagous. Overwintering beetles emerged at the end of May or earlier, then feeding and mating on the host plants. The highest level of adult activity was observed at the end of June. Larvae were endophagous within the flower heads. In July and August, the larvae pupated within inflorescences in a pupation cell. Adults exited the cells at the end of August and did not hibernate on the host plants. Sometimes, larvae and imagines of a new generation were found outside the flower heads in chambers constructed on the stems.

## Introduction

The weevil genus *Larinus* Dejean, 1821 belongs into the tribe Lixini Schoenherr, 1823 and is represented by ca. 180 species (Csiki 1934; Ter-Minassian 1967; Gültekin 2006) of which more than 110 are known in the Palaearctic (Gültekin and Fremuth 2013; Gültekin and Shahreyary-Nejad 2015). A further 40 species are recorded from the Ethiopian region, only three species from the Oriental region, four (introduced species) from the Nearctic region (McClay 1988; Gültekin 2006), and one in New Zealand (Woodburn and Briese 1996; Gültekin 2006). The valid systematic position of this genus has been assigned for Palaearctic species in the Catalogue of Palaearctic Coleoptera (Gültekin and Fremuth 2013). The genus is divided into four subgenera: *Cryphopus* Petri, 1907; *Larinus* Dejean, 1821; *Larinomesius* Reitter, 1924; and *Phyllonomeus* Gistel, 1856 (Gültekin and Fremuth 2013). Knowledge of the morphology of immature stages in *Larinus* is incomplete in comparison to the total number of species in this genus and to the importance of several species as potential biological control agents against weeds (Nikulina et al. 2004; Seastedt et al. 2007).

The species *Larinus vulpes* (Olivier, 1807) belongs to the nominotypical subgenus Larinus, which includes 35 species in the Palaearctic region (Gültekin and Fremuth 2013; Gültekin and Shahreyary-Nejad 2015), and is distributed in the western Palaearctic, east Siberia and central Asia (Gültekin and Podlussány 2012; Gültekin and Fremuth 2013). The most northern area of its range is Kungur, Russia (56–57°N) (Dedyukhin 2011). The life cycle of *L. vulpes* is associated only with globe thistles, the genus *Echinops* L. (Asteraceae). Similar to other *Larinus* species, adults feed on the leaves and stems. Eggs, larvae, and pupae develop in the inflorescences. Imagines of a new generation hibernate outside the host plants. Circumstantial observations of reproduction and preimaginal development of *L. vulpes* were reported by Fabre (1922; as *Larinus maculosus* Schoenherr, 1832). Next, Volovnik (1996, 2016) provided sufficient details on the biology of this species. The immature stages of this species have never been morphologically described.

Some species from the genus *Echinops* are very important invasive weeds (Czarapata 2005; Reddy et al. 2008) and also have medicinal uses (Murch et al. 2003; Eram et al. 2013; Parhat et al. 2014). The globe thistles are nectar (Wroblewska et al. 1993; Jabłoński and Kołtowski 2005) and ornamental plants (Wiersema and León 2016), a potential source of natural insecticide (Gemechu et al. 2013; Liu et al. 2013), molluscicid (Hymete et al. 2005), and energetic oil (Horn et al. 2008). The root extract of *E. giganteus* A. Rich. is used as a mosquito repellent (Karunamoorthi and Hailu 2014) and also as perfume (The Green Vision 2017), and these roots are used as dietary spice (Stève et al. 2016). Because of their deep roots, the globe thistles are widely used for mechanical stabilisation of banks, ravines, and slopes (Chopik et al. 1983). Therefore, there are strong arguments for a detailed investigation of the weevil *L. vulpes* and then promote the use of larvae of *L. vulpes* as potential biological control agents against this plant. The knowledge of bionomy of immature stages of *Larinus* species is also important for further taxonomic studies at different levels and for effective protection of endangered species. In this paper, we describe the immature stages of *L. vulpes* and provide details of its life history based on field observations in Ukraine.

## Materials and methods

### Insect collection and laboratory breeding

The material used to describe the immature stages was collected and field observations were conducted in Ukraine in the following localities:

1) The eastern shore of Molochnyi Estuary between the two villages of Altagir (= Bogatyr) and Radyvonivka (46°38'29"N, 35°16'59"E). Altitude: up to 20 m a. s. l. Bedrock: loess loam with herbaceous covering. Dominant plant species: *Agropyron pectinatum* (M. Bieb.) P. Beauv., *Festuca valesiaca* Schleich. ex Gaudin, *Koeleria cristata* (Ledeb.) Schult., *Artemisia marschalliana* Spreng., *Ephedra distachya* L., and *Helichrysum arenarium* (L.) Moench, 1794 (Kolomiychuk and Vynokurov 2016). Globe thistle *Echinops ruthenicus* M. Bieb. usually grows along the top of the slope.

2) Man-made forest near the Kamyana Mohyla Reserve (46°57'01"N, 35°28'12"E). Altitude: up to 10 m a. s. l. Bedrock: sandy chernozem. Dominant plant species: *Robinia pseudoacacia* L. and *Morus nigra* L., with herbaceous plants (i.e., *Echinops ruthenicus*, *Centaurea adpressa* Ledeb. ex Steud., *Melilotus albus* Medik.) in the clearings.

3) NE Cyrilivska Spit located between the Sea of Azov and Molochnyi Estuary (46°25'12"N, 35°25'09"E). Altitude: 5 m a. s. l. Bedrock: flats of quartz sand, shells and mud. Habitats: salty grasslands with dominant herbaceous species *Elytrigia elongata* (Host) Nevski, *Puccinellia distans* (Jacq.) Parl., *Aeluropus littoralis* (Gouan) Parl., 1850 and *Juncus gerardii* Loisel.; coastal communities with *Phragmites australis* (Cav.) Trin. ex Steud., *Bolboschoenus maritimus* (L.) Palla and *Juncus maritimus* Lam. (Kolomiychuk and Vynokurov 2016). Globe thistle *Echinops ruthenicus* is locally distributed between the central road and the coast of the estuary (Fig. 21).

In the above-mentioned localities, life cycle, including feeding of adults, oviposition, and early development of larvae were observed directly during the vegetation growing seasons of *Echinops ruthenicus* and *E. sphaerocephalus* L. in the time period 2012–2016.

The compound flower heads of globe thistles consist of simple capitula, each of which has only one floret. These primary capitula are aggregated in globose secondary capitula (Kadereit and Jeffrey 2007). For convenience, below, we shall name as capitula only secondary ones.

The second author collected all larvae and pupae of *L. vulpes* within inflorescence. Some inflorescences (n = 42) were dissected to investigate preimaginal development, and a further 250 were dissected to determine the quantity of preimaginal specimens of *L. vulpes* within an inflorescence. All photographs in the field were taken with digital cameras, a Nikon Coolpix 4600 and a Canon PowerShot SX500 IS.

Laboratory observations were conducted in Melitopol, Ukraine (46°50’N, 35°22’E). The dry inflorescences (n = 7) with developing mature larvae or pupae were placed into cardboard boxes. A small hole was opened in every inflorescence for possible observations of insect development. Measurements of flower head were performed with a slide caliper and ocular micrometre. The size of flower heads was determined at the greatest diameter.

Geographical distribution and phenology were studied from several entomological collections, specifically: Schmalhausen Institute of Zoology of National Academy of Sciences of Ukraine (Kyiv), TG Shevchenko Kyiv National University Zoological Museum, Zoological Institute of Russian Academy of Sciences (St. Petersburg), VN Karazin Kharkiv National University Museum of Natural History, and Igor Maltzevs’ private collection (Odessa). In total, more than 130 pinned specimens were studied.

### Morphological descriptions

Part of the larval and pupal material was preserved in Pampel fixation liquid (see Skuhrovec et al. 2015) and used for the morphological descriptions. These specimens are deposited in the collection of Group Function of Invertebrate and Plant Biodiversity in Agro-Ecosystems of the Crop Research Institute (Prague, Czech Republic). The collectors identified the plants. To prepare slides, we followed May (1994).

The observations and measurements were conducted using a light microscope with calibrated oculars (Olympus BX 40, SZ 11, and Nikon Eclipse 80i). The following characteristics were measured for each larva: head width, length of the body (larvae fixed in a C-shape were measured in the middle of the segments in lateral view), and width of the body in the widest place (i.e., meso- and metathorax). For the pupae, the length and the width at the widest place were measured.

Drawings were created with a drawing tube on a light microscope and edited by the programs Adobe Photoshop 10, Corel Photo-Paint X7, and GIMP 2.

We used the terms and abbreviations for the setae of the mature larva and pupa found in Scherf (1964), May (1977, 1994) and Marvaldi (1998, 1999). The numbers of setae of the bilateral structures are given for one side.

The counts of some of the setae on the epipharynx (particularly *ams* and *mes*) have not been completely resolved. According to Marvaldi (1998, 1999), the standard status of the epipharynx in weevils is 2 *ams* and 3 *mes*; however, when the position of the distal *mes* is very close to the anterior margin, it appears as *ams.* The final decision was to add this problematic seta to the latter group (*ams*), and the position of this seta is similar to that in other genera, e.g., in *Coniocleonus* Motschulsky, 1860 or *Tychius* Germar, 1817. We did not follow Stejskal et al. (2014) and Skuhrovec et al. (2014) who accepted the standard status in weevils and counted the seta as *mes*, but we followed Trnka et al. (2015) and Skuhrovec et al. (2015), e.g., in *Adosomus* Faust, 1904 or *Sibinia* Germar, 1817. The thoracic spiracle was located on the prothorax near the boundary of the prothorax and mesothorax, as shown in the drawing (see Fig. 10), but this spiracle is of mesothoracic origin (Marvaldi et al. 2002; Marvaldi 2003).

## Results and discussion

### Morphology of immature stages

#### Larinus (Larinus) vulpes

Taxon classificationAnimaliaColeopteraCurculionidae

(Olivier, 1807)

##### Material examined.

**UKRAINE**: Cyrilivska Spit (locality 3 in Materials and methods), 12–29.viii.2015, 15 larvae, 16 pupae (5♂♂, 11♀♀), leg. S Volovnik.

##### Description of mature larva.

*Measurements* (in mm, n = 15). Body length: 13.4–21.2 (mean 18.1). Body width: (meso- and metathorax) 4.03–5.01 (mean 4.46). Head width: 1.99–2.29 (mean 2.14).

*General.* Body stocky, slightly curved, rounded in cross section (Fig. 7). Cuticle densely spiculate (Figs 8–9).

*Colouration.* Head light brown or brown with a distinct pale pattern around the frontal and epicranial sutures (Fig. 7). All thoracic and abdominal segments are dark yellow with a light brown, elongate stripe on the dorsum of the pronotum (Fig. 7).

*Vestiture.* Setae on body thin, short, light yellow or orange (Figs 7–9).

**Figure 1.**
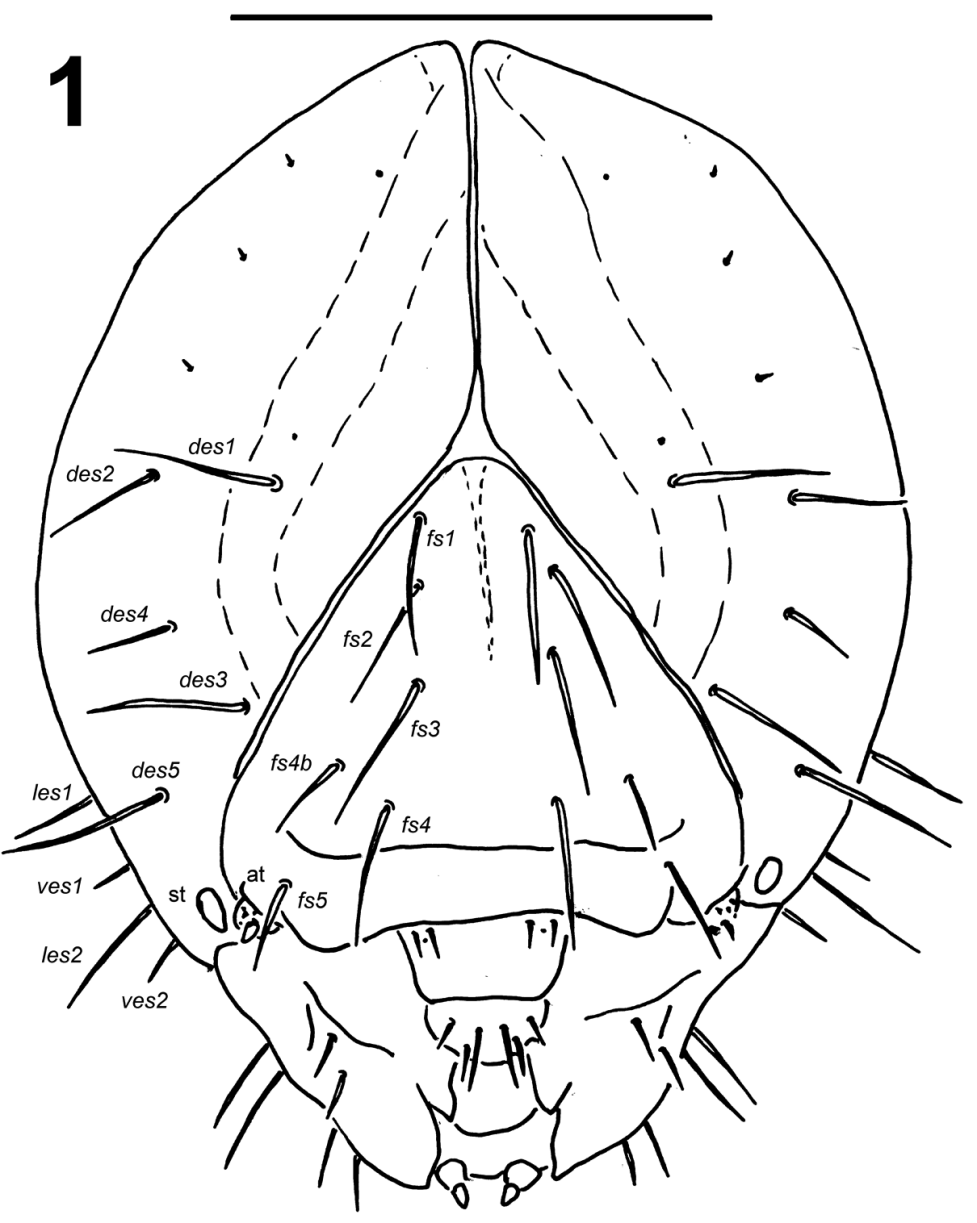
*Larinus vulpes* mature larva head, frontal view. Abbreviations: *des* – dorsal epicranial s., *fs* – frontal epicranial s., *les* – lateral epicranial s., *ves* – ventral epicranial s., at – antenna, st – stemma. Scale bar 1 mm.

*Head capsule* (Fig. 1). Head suboval, slightly longer than wide, endocarinal line weak, but long as a half-length of frons. Frontal sutures distinct, wide, and extended to the antennae. Single anterior stemma (st) distinct, in the form of a slightly pigmented spot. *Des_1_* and *des_2_* located in the upper part of the central part of the epicranium, *des_1_* near the middle part of the epicranium and *des_2_* near the side of the epicranium, *des_3_* located anteriorly near the frontal suture, *des_4_* located in the central part of the epicranium, *des_5_* located anterolaterally; all *des* long, nearly subequal in length, except *des_4_* distinctly shorter (Fig. 1). *Fs_1_*, *fs_2_* and *fs_3_* placed medially, *fs_4_* located anteromedially, *fs_4b_* located laterally close to *fs_4_*; and *fs_5_* located anterolaterally, close to the epistoma; all setae very long to long, only *fs_4b_* and *fs_5_* medium, distinctly shorter than very long *fs_1-4_* (Fig. 1). *Les_1–2_* and *ves_1–2_* very long, as long as *des_5_*. Epicranial area with two sensilla, one upper *des_1_* and the second in upper part of posterior; and also with 3 *pes* in line with upper *des_2_*.

*Antennae* located at the end of the frontal suture on each side, membranous and slightly convex basal article bearing one conical sensorium, relatively long; basal membranous article with 5 sensilla, different in both shape and length (Fig. 4).

**Figures 2–3.**
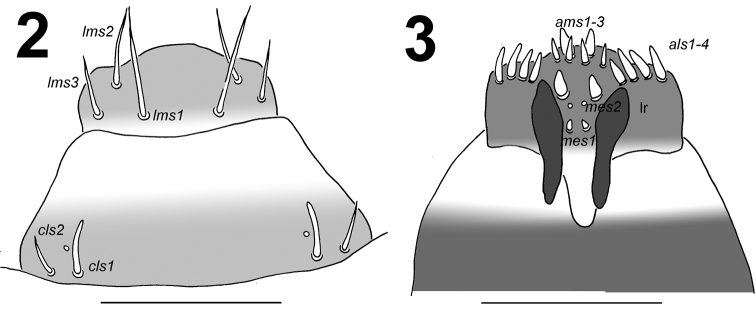
*Larinus vulpes* mature larva, mouth parts. **2** Labrum and clypeus **3** Epipharynx. Abbreviations: *ams* – anteromedial s., *als* – anteriolateral s., *cls* – clypeal s., *mes* – median s., *lms* – labral s., lr – labral rods. Scale bar 0.2 mm.

*Clypeus* (Fig. 2) trapezoid-shaped, approximately twice as wide as long, with two relatively long *cls*, *cls_2_* slightly shorter than *cls_1_*, localized posterolaterally and 1 sensillum located close to *cls_1_*; anterior margin concave.

*Mouth parts.* Labrum (Fig. 2) approximately twice as wide as long, with 3 pairs of piliform *lms*, of different lengths; *lms_3_* distinctly shorter than very long *lms_1_* and long *lms_2_*; *lms_1_* located close to the margin with clypeus, *lms_2_* located anteromedially, and *lms_3_* located anterolaterally; anterior margin bisinuate. Epipharynx (Fig. 3) with 4 blunt, finger-like *als*, unequal in length, *als_1–2_* shorter than *als_3–4_*; 3 *ams*: *ams_1_* and *ams_2_* distinctly thinner than *ams_3_*; *ams_1_* and *ams_2_* piliform, *ams_3_* blunt, finger-like; 2 *mes* and one sensillum close to *mes_2_*; *mes_1_* distinctly smaller than *mes_2_*, both located close to lr; labral rods (lr) elongated, slightly diverging on distal half. Mandibles (Fig. 5) relatively broad, bifid, teeth of unequal height; slightly truncate; both *mds* relatively long, piliform. Maxilla (Fig. 6) stipes with 2 *stps*, 2 *pfs*, and 1 *mbs*; both *stps* and *pfs_2_* very long and 1.5 times longer than long *pfs_1_*, *mbs* very short; mala with 9 bacilliform *dms_1-9_*; 5 short *vms_1-5_*, in two sizes: 2 *vms* short, and 3 very short; all *vms* distinctly shorter than *dms.* Maxillary palpi with two palpomeres; basal palpomere with 1 short *mxps* and two sensilla; length ratio of basal and distal palpomeres: 1:0.9; distal palpomere with one sensillum and a group of conical, apical sensorial papillae. Praelabium (Fig. 6) heart-shaped, with 1 relatively long *prms*; ligula with sinuate margin and 2 piliform very short *ligs*, unequal in length; premental sclerite V-shaped clearly visible. Labial palpi with two palpomeres; length ratio of basal and distal palpomeres: 1:0.9; distal palpomere with one sensillum and short, apical sensorial papillae; basal palpomere with 1 ventral sensillum. Postlabium (Fig. 6) with 4 *pms*, 1 *pms* located anteriorly, and 3 *pms* laterally; very long, almost of equal length; surface of postlabium densely covered by distinct asperities.

*Thorax.* Prothorax larger than meso- and metathorax. Meso- and metathorax distinctly wider than abdominal segments I–IV. Spiracle unicameral. Cuticle densely spiculate and with distinct thorn-like cuticular processes, primarily on dorsal parts but also on pleural parts (Fig. 7). Prothorax (Fig. 10) with ca. 30–35 relatively long to short *prns* unequal in length, 25 on pigmented pronotal sclerite, which is subdivided medially into two triangular plates, next 5–10 *prns* placed below; 20 relatively long *ps* also on pigmented sclerite, and 12 relatively long *eus.* Mesothorax (Fig. 10) with 3 short *prs*; 13 relatively long to short *pds*; 6–7 relatively long to short *as*; 6 relatively long to short *ss* on pigmented sclerite; 6–9 relatively long to short *eps* on pigmented sclerite; 14 relatively long to short *ps* on pigmented sclerite and 12 relatively long to long *eus.* Chaetotaxy of meso- and metathorax (Fig. 10) almost identical, but some specimens partly variable in the exact count of setae. Each pedal area of the thoracic segments well separated and pigmented, with 10 long *pda* on pigmented sclerite, unequal in length.

*Abdomen.* Abdominal segments I–IV of almost equal length, subsequent abdominal segments decreasing gradually to the terminal parts of the body. Abdominal segment X reduced to four anal lobes of unequal size, the dorsal being distinctly the largest, the lateral pair equal in size, and the ventral lobe very small. Anus located terminally. Spiracles unicameral, the eight abdominal spiracles located laterally, close to the anterior margin of abdominal segments I–VIII. Cuticle also densely spiculate and with distinct thorn-like cuticular processes, primarily on dorsal parts but also on pleural parts (Figs 8–9). Abdominal segments I–VII (Figs 11–12) with 2 relatively long to short *prs*; 13 relatively long to short *pds*, 10 *pds* in line, and 3 *pds* in the below part partly anteriorly; 7 relatively long to short *ss*, 5 *ss* under *pds* (abdominal segment VII only with 3 setae), and 2 *ss* in below part of dorsal lobe; 13 (10–14) relatively long to short *eps* on pigmented sclerite (only on abdominal segments I–II); 9 relatively long *ps* of unequal length; 2 short *lsts* and 2 short *eus.* Abdominal segment VIII (Fig. 12) with 2 relatively long *prs*; 10 relatively long to long *pds* in line; 2 relatively long *ss* in below part of dorsal lobe; 13 relatively long to short *eps*; 9 relatively long *ps* of unequal length; 2 short *lsts* and 2 short *eus.* Abdominal segment IX (Fig. 12) with 7 *ds* (6 long *ds* near posterior margin, and 1 short *ds* medially); 13 relatively long to long *ps*; and 2 relatively long and 2 short *sts.* Abdominal segment X (Fig. 12) with 2 very short setae (*ts*) on each lateral anal lobe, and 1 very short seta (*ts*) on dorsal anal lobe.

##### Description of pupa.

*Measurements* (in mm; 5 ♂♂, 11 ♀♀). Body length: ♂ 11.8–13.2 (mean 12.5), ♀ 12.6–15.2 (mean 14.0). Body width: ♂ 6.5–7.8 (mean 6.8), ♀ 7.0–8.20 (mean 7.5). Thorax width: ♂ 4.6–4.9 (mean 4.7), ♀ 4.8–5.1 (mean 4.9). Head width: ♂ 1.8–2.0, ♀ 1.9–2.2.

*Colouration.* All thoracic and abdominal segments light yellow or greenish-white. Cuticle smooth, except thorn-like processes on abdominal segments III–VIII.

*Morphology* (Figs 13–20). Body moderately slender and elongated. Rostrum long, approximately 2.5 times as long as wide, extended to mesocoxae. Antennae relatively long and slender. Pronotum 2.5 times as wide as long. Meso- and metanotum of equal length. Abdominal segments I–V of equal length, abdominal segments V–VII diminish gradually, abdominal segment VIII almost semi-circular, and abdominal segment IX distinctly smaller than other segments. Urogomphi very short, almond-shaped with acute sclerotised apexes. Spiracles placed dorso-laterally; 5 pairs functional on abdominal segments I–V and one atrophied on abdominal segment VI, on next abdominal segments spiracles invisible. Sexual dimorphism visible in the structure of abdominal segment IX: gonotheca of ♀ divided (Fig. 19), ♂ undivided (Fig. 20).

*Chaetotaxy* (Figs 13–20). Setae distinct, of different length, light brown. Head capsule includes 1 vertical seta (*vs*); 3 super-orbital setae (*sos_1–3_*) (equal in length); 1 orbital seta (*os*) and 4 post-antennal setae (*pas_1–4_*) (equal in length). Rostrum with 8 rostral setae (*rs_1–8_*) (different in length); *rs_1–4_* located apically, *rs_5–7_* latero-apically, and *rs_8_* latero-medially. Mandibular theca with 1 epistomal seta (*es*). Setae on head capsule and rostrum straight or slightly curved, shorter than setae on pronotum. Pronotum with many setae, which created a characteristic pattern that consisted of: 1 super apical seta (*sas*), 4 super lateral setae (*sls_1–4_*) (equal in length), distributed along a horizontal line, 3 discal setae (*ds_1–3_*) equal in length, forming a group medially, and 21 posterolateral setae (*pls_1–21_*) (different in length) of which: *pls_1–11_* located along posterior margin of pronotum and next 10 setae (*pls_12–21_*) form groups ventrally. Lateral setae in two groups; *ls_1–5_* located more or less along margin of pronotum, next five (*ls_6–10_*) form a group (sometimes covered by antennae). Setae on pronotum different in length: *pls_1–11_* the longest, *ds_1–3_* the shortest (Fig. 16). Prolegs with 5 trochanter setae (*trs_1–5_*). Mesonotum with 14 setae (*d_1–14_*): *d_1–3_* located anteromedially, *d_4_* posteromedially and *d_5–14_* mediolaterally. Metanotum with 14 setae (*d_1–14_*): *d_1–3_* located anteromedially, *d_4–14_* form a line medially (Fig. 16). Apex of femora with 3 or 4 long femoral setae (*fes*) (Fig. 18). Setae on abdominal segments various in size, sometimes replaced by thorn-like cuticular processes; a reason the exact number of setae remained difficult to precisely determine. Approximately 40 setae are on dorsal parts of each of abdominal segments I and II, and approximately 30 on each of abdominal segments III–VIII. Generally: 4–6 very short setae placed along anterior margin of each segment, next 20 long and short setae (or ca. 10 long setae and ca. 10 thorn-like cuticular processes) form a regular, horizontal line on median parts; next approximately 15 thin setae form groups dorsolaterally on each segment. Some small setae are replaced by thorn-like cuticular processes, which increase gradually from abdominal segment III to VII. Cuticular processes on abdominal segment VIII distinctly smaller than those on abdominal segments VI and VII. Abdominal segment VIII with 5 small setae along anterior margin, 6 medium size setae and some small cuticular processes medially. Abdominal segments I–VIII with approximately 20 thin setae (*l_1–20_*) laterally (Fig. 18); ventral parts of abdominal segments I–VIII with 3 setae located medially or medio-laterally. Abdominal segment IX with 5 short setae, located ventrally along anterior margin of segment, and next single seta on gonothecae. External parts of urogomphi are densely covered by thin setae.

**Figures 4–5.**
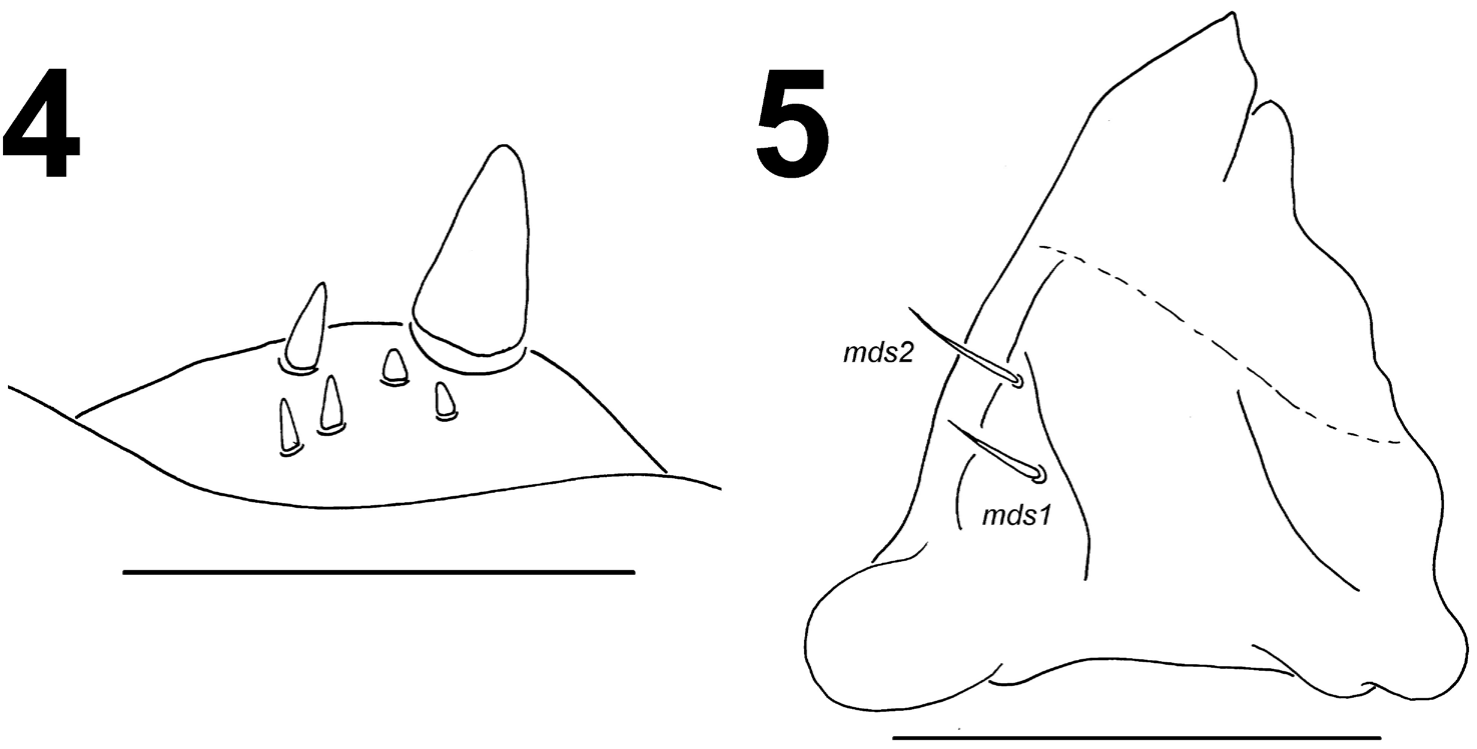
*Larinus vulpes* mature larva, antenna, and mouth parts. **4** Antenna **5** Right mandible (*mds* – mandible dorsal s.). Scale bars 0.05 mm (**4**) and 0.5 mm (**5**).

**Figure 6.**
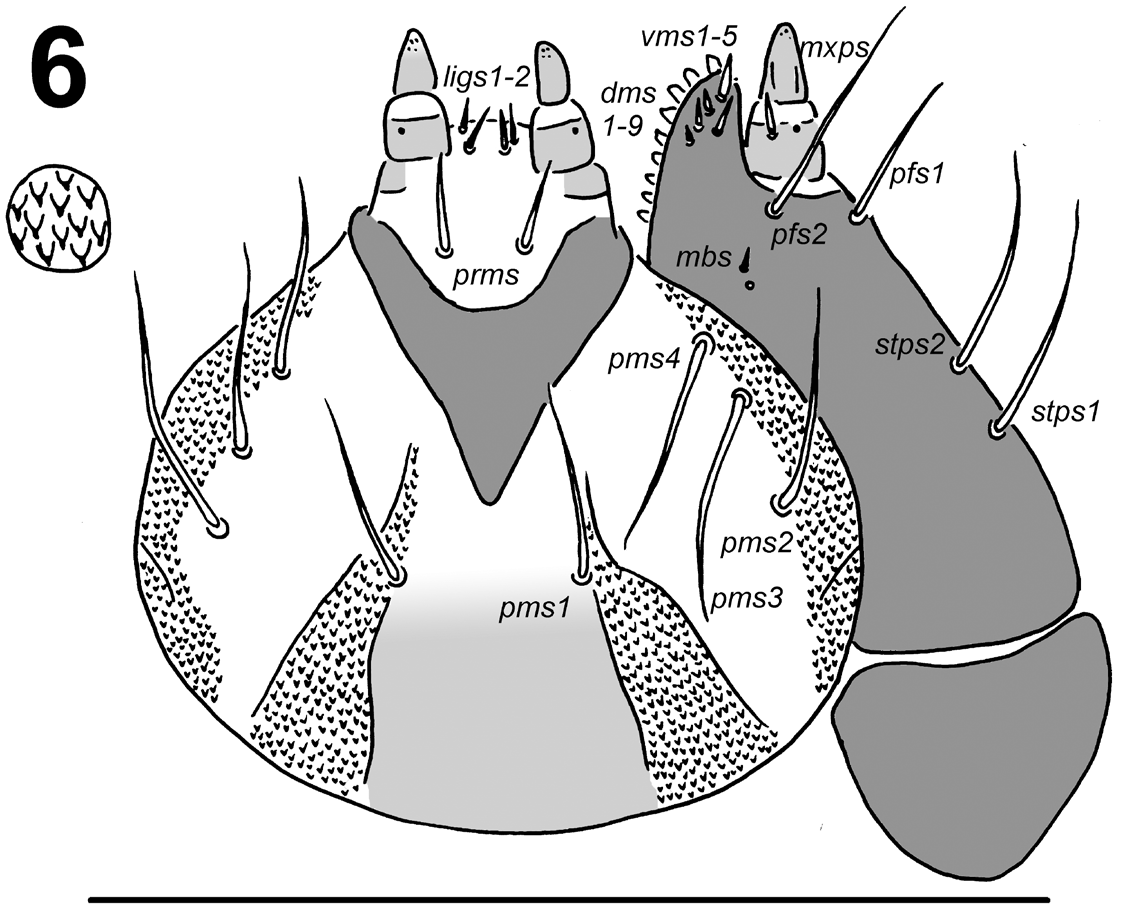
*Larinus vulpes* larval mouthparts, maxillolabial complex, ventral view: right maxilla. Abbreviations: *dms* – dorsal malar s., *vms* – ventral malar s., *mpxs* – maxillary palps s., *mbs* – basioventral s., *pfs* – palpiferal s., *stps* – stipital s.), prementum and postmentum, ventral view (*prms* – premental s., *pms* – postmental s., *ligs* – ligular s.). Scale bar 1 mm.

**Figure 7–9.**
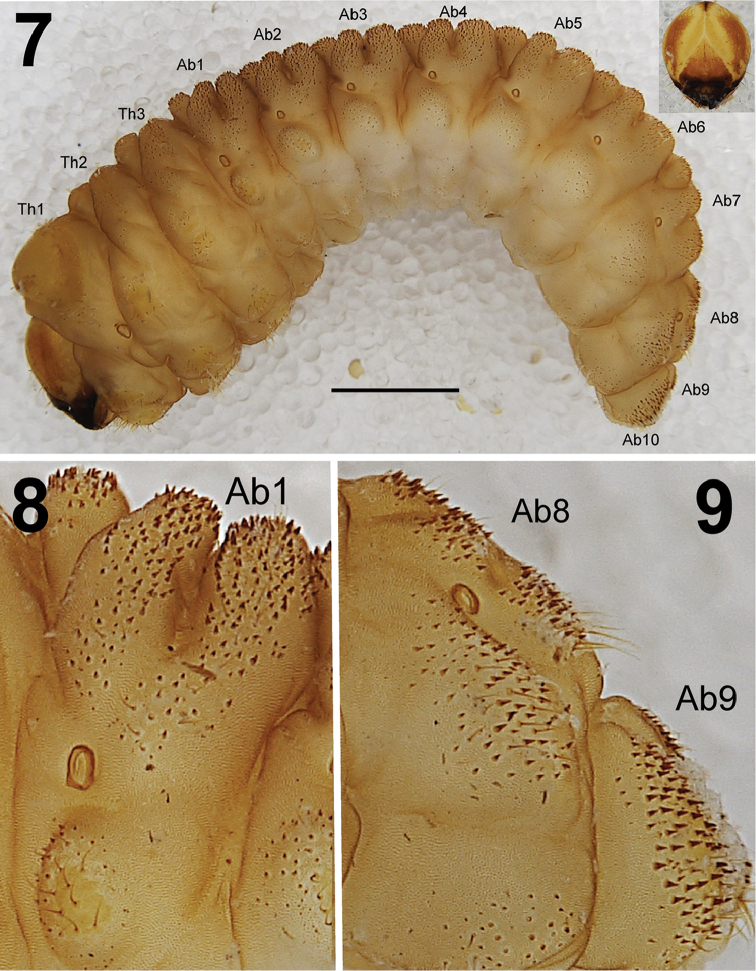
*Larinus vulpes* mature larva. **7** Habitus, lateral view. Scale bar: 2 mm. **8** Detail lateral view on dorsum of abdominal segment I with densely spiculate cuticle **9** Detail lateral view on dorsum of abdominal segment VIII–IX.

**Figures 10–12.**
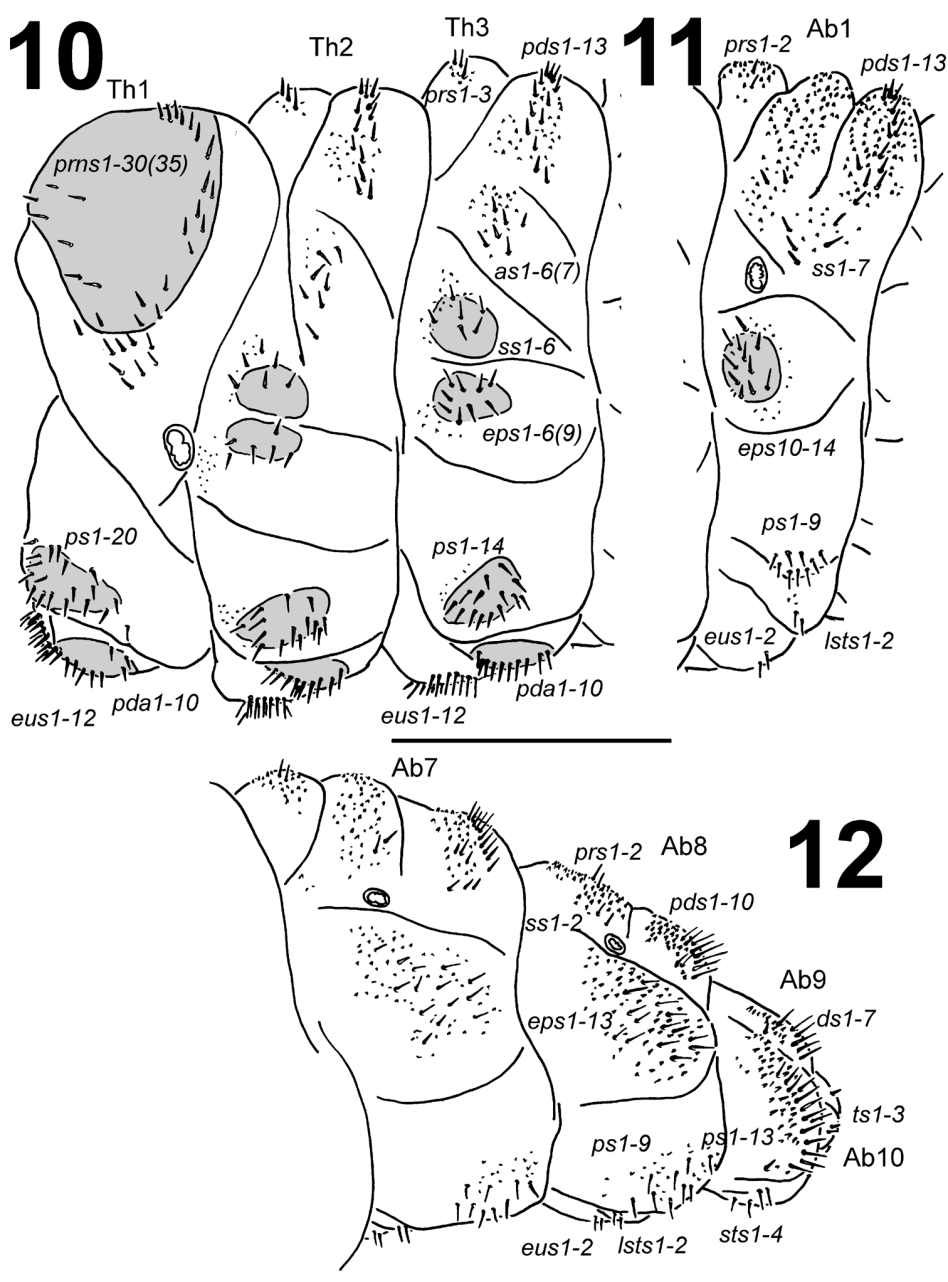
*Larinus vulpes* mature larva, habitus. **10** Lateral view of thoracic segments **11** Lateral view of abdominal segment I **12** Lateral view of abdominal segments VII-X. Abbreviations: *prns* – pronotal s., *prs* – prodorsal s., *pds* – postdorsal s., *as* – alar s., *ss* – spiracular s., *eps* – epipleural s., *ps* – pleural s., *pda* – pedal s., *lsts* – laterosternal s., *eus* – eusternal s., *ds* – dorsal s., *sts* – sternal s., Th1-3 – number of thoracic segments, Ab1-10 – number of abdominal seg. Scale bar 2 mm.

**Figures 13–15.**
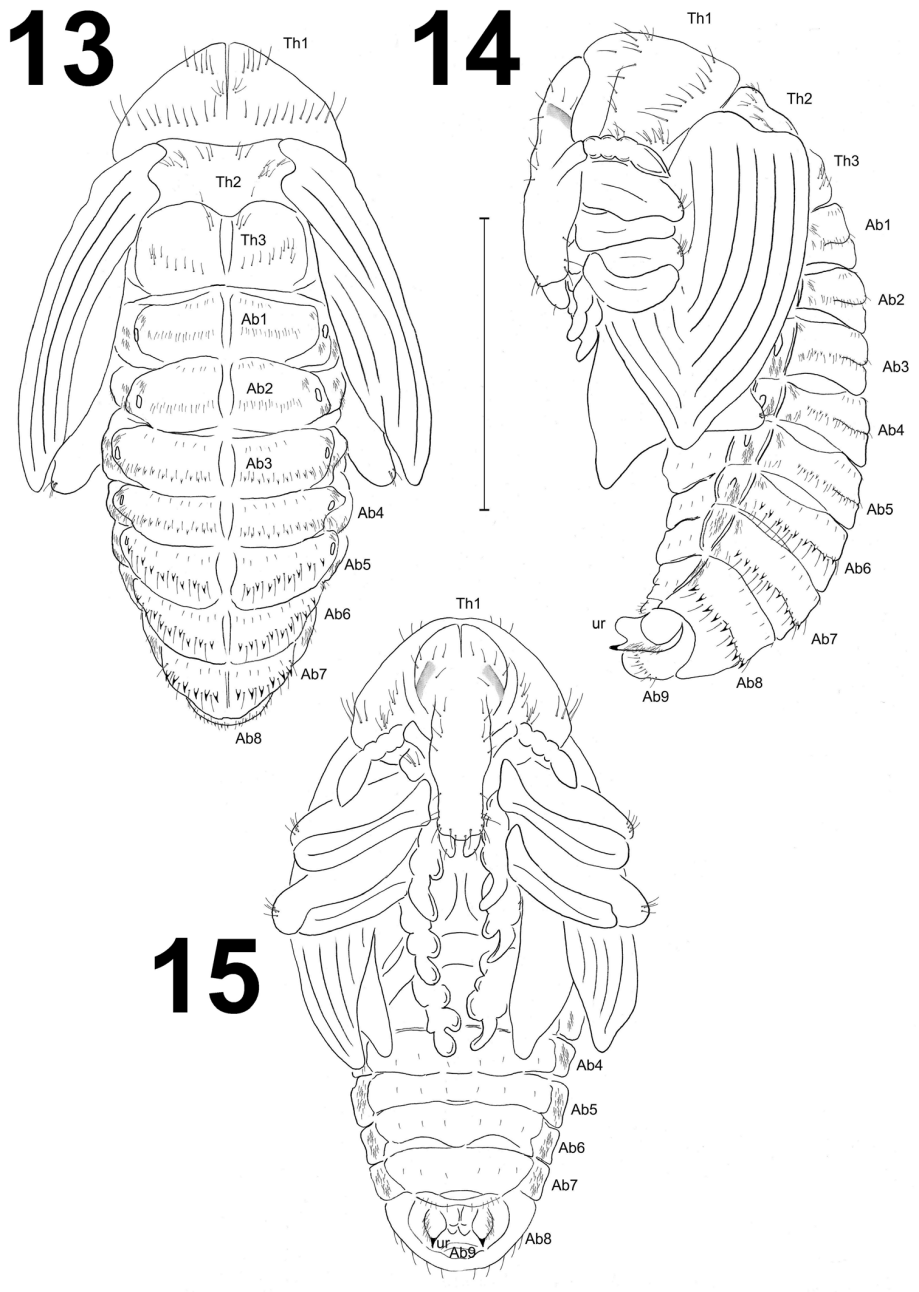
*Larinus vulpes* pupa habitus. **13** Dorsal view **14** Lateral view **15** Ventral view. Abbreviations: Ab1-9 – number of abdominal segments, Th1-3 – number of thoracic segments, ur – urogomphi. Scale bar 5 mm.

**Figures 16–20.**
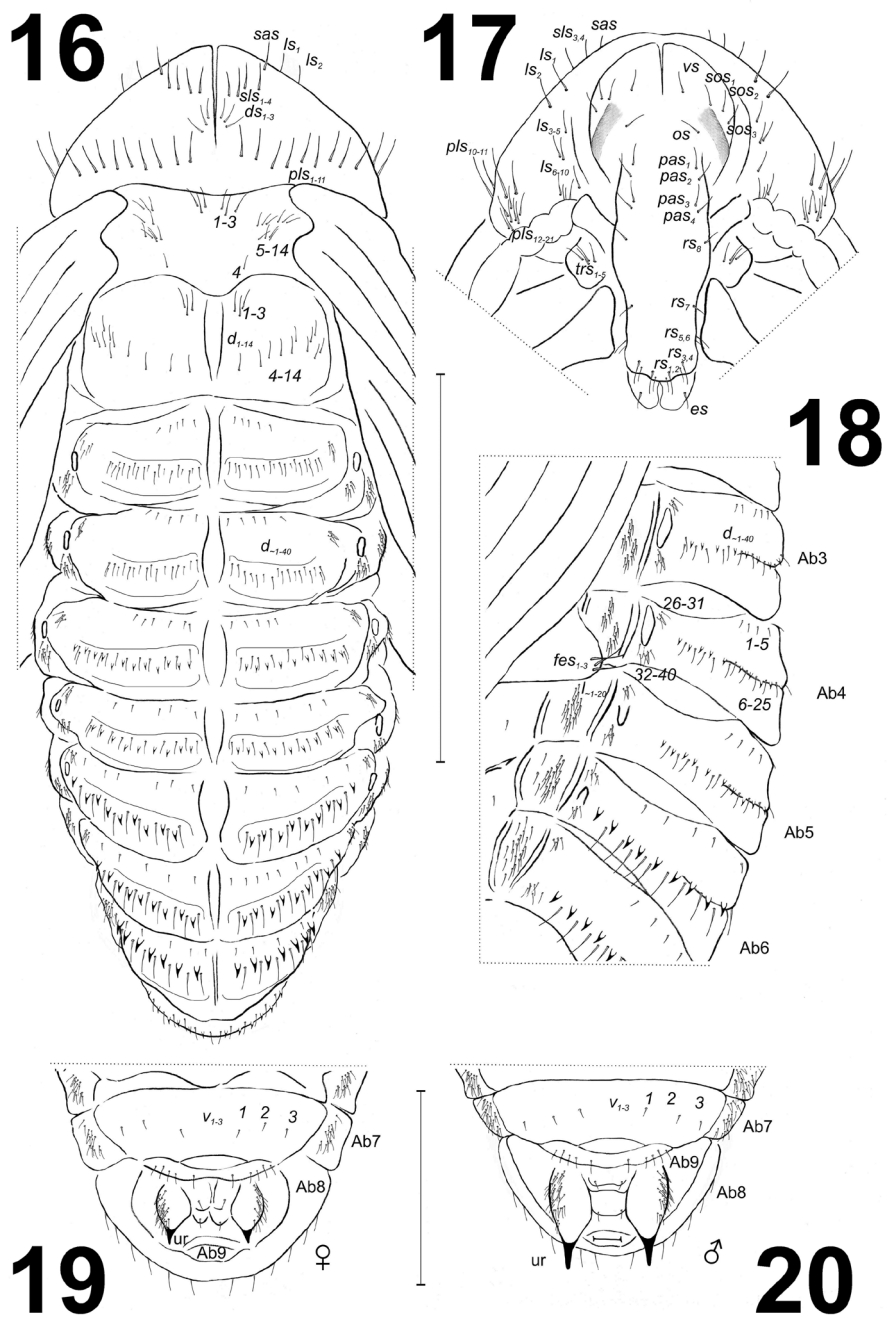
*Larinus vulpes* pupa chaetotaxy. **16** Dorsal view **17** Head and rostrum **18** Lateral view of abdominal segments 3–7 **19** Ventral view of lasts abdominal segments of female **20** Ventral view of lasts abdominal segments of male. Abbreviations: Ab1-9 – number of abdominal segments, Th1-3 – number of thoracic segments, ur – urogomphies. Setae: *sas* – super apical, *l*, *ls* – lateral, *sls* – super lateral, *d* – dorsal, *ds* – discal, *pls* – posterolateral, *v* – ventral, *vs* – vertical, *sos* – super orbital, *os* – orbital, *pas* – postantennal, *rs* – rostral, *es* – epistomal, *fes* – femoral. Scale bars 5 mm (**16–18**), and 2 mm (**19–20**).

##### Comparison with larvae of other *Larinus* species.

To date, larvae of 16 *Larinus* species have been described (Gardner 1934; Scherf 1964; Lee and Morimoto 1988; Nikulina et al. 2004; Zotov 2009a, 2010; Gosik and Skuhrovec 2011; Nikulina and Gültekin 2014), while detailed descriptions of the pupae are known for only 8 *Larinus* species (Zotov 2009a, 2010; Gosik and Skuhrovec 2011; Nikulina and Gültekin 2014). The comparison with previously described immatures of some other species, primarily of L. (Phyllonomeus) saussureae Marshall, 1924 (Gardner 1934), L. (Phyllonomeus) carlinae (Olivier, 1807) (as *L. planus* F.) and L. (Phyllonomeus) iaceae (Fabricius, 1775) (both in Scherf 1964), was somewhat problematic because of missing details of chaetotaxy and/or absence of quality drawings; therefore, a comparison of these three species with other known *Larinus* species was not possible to the level of detail required to incorporate them in the key (see Key to the immature stages of the *Larinus*). Lee and Morimoto (1988) provide a general larval description of the genus *Larinus* based on two species: L. (Phyllonomeus) latissimus Roelofs, 1873 and L. (Phyllonomeus) meleagris Petri, 1907. However, they did not present any differences between these two species (see aggregation of both species in the key at dichotomy 12).

According to May (1993), the increased number of *pds* on the meso- and metathorax and abdominal segments I–VII and of setae on the epipharyngeal lining (*als*) (i.e., more than the most frequent number of setae in weevils) are diagnostic of the mature larva of the subfamily Lixinae. The following descriptions of mature larvae from the tribe Lixini confirmed this diagnosis: genus *Larinus* (Scherf 1964; Lee and Morimoto 1988; Nikulina et al. 2004; Zotov 2009a, 2010; Gosik and Skuhrovec 2011; Nikulina and Gültekin 2014); genus *Lixus* (Scherf 1964; Lee and Morimoto 1988; May 1994; Nikulina 2001, 2007; Zotov 2009a, b; Nikulina and Gültekin 2011; Gosik and Wanat 2014; Skuhrovec and Volovnik 2015; Trnka et al. 2016); and *Rhinocyllus conicus* (May 1994), in addition to descriptions of all known species from the tribe Cleonini (Zotov 2011; Stejskal et al. 2014; Trnka et al. 2015). For a proper comparison of both tribes, including a key and detailed generic studies, further descriptions of immature stages of several Cleonini would be required.

Gosik and Wanat (2014), in a precise general description of the larvae of the tribe Lixini, summarized the tribe by 16 character sets (for details, see Gosik and Wanat 2014), but some of these characters (primarily chaetotaxy on the body) do not correspond exactly with most *Larinus* species from the nominotypical subgenus, including the recently described *L. vulpes*. The species from the subgenus Larinus (except *L. idoneus* Gyllenhal, 1835 and *L. latus* (Herbst, 1783)) had very high counts of larval body setae; e.g., pronotum with more than 25 setae and postdorsum on meso- and metathorax and also on abdominal segments I–VII with more than 12 setae (see details in Key to the immature stages of the *Larinus* and Table 1). The pupal number of setae was identical to that of all known pupae of species from the subgenus Larinus (except *L. idoneus*) with a pronotum with 25 or even more setae (see details in Key to the immature stages of the *Larinus* and Table 2). Morphological characters of larvae and pupae distinctly separated the subgenus Larinus from the other subgenera *Phyllonomeus* Gistel, 1856 and *Larinomesius* Reitter, 1924. Only two species (*L. idoneus* and *L. latus*) from the nominotypical subgenus did not correspond with the described chaetotaxy, which could be explained considering three hypotheses: (1) the nominotypical subgenus can be divided into two distinct groups, (2) these two species do not belong in this subgenus, or (3) these species show a peculiar autapomorphy; a change in a setal number can be a mere convergence (or coincidence). To solve this problem further morphological and molecular studies would be necessary.

The immature stages of *L. vulpes* had the closest affinity to the larvae of L. (L.) inaequalicollis Capiomont, 1874 and L. (L.) capsulatus Gültekin, 2008 based on five larval morphological characters: (1) frons with 6 or 7 *fs*; (2) postlabium with 4 or 5 setae; (3) stipes with 2 long *sts*; (4) prodorsum on meso- and metathorax with 3 *prs*; and (5) dorsal part of body distinctly spiculate; and two pupal morphological characters: (6) cuticle around setae dark-pigmented, visible spots formed; and (7) rostrum with 3 *pas* and only 1 *rs.* The primary differences between *L. vulpes* and *L. inaequalicollis* were as follow (see key to the immature stages of the *Larinus*): postepicranial setae *pes_1_*–*pes_2_* distinct (*versus L. inaequalicollis* very small, indistinct); frons with 6 *fs* (*versus* with 7 *fs*); endocarina not distinct, its length is as half-length of frons or less (*versus* distinct, massive, approximately 2/3 the length of frons), and ligula with 2 very thin *ligs* (*versus* with 1 micro *ligs* and two sensillae). The primary differences between *L. vulpes* and *L. capsulatus* were are as follows (see key to the immature stages of the *Larinus*): postlabium with 4 setae (*versus L. capsulatus* with 5 setae); meso- and metathorax with 6–7 *as*, 6 *ss* and 6–9 *eps* (*versus* with 4 *as*, 4 *ss* and 5 *eps*); abdominal segments I–VII with fewer than 14 *pds* and more than 10 *eps* (*versus* more than 15 *pds* and 8 or fewer *eps*); and lateral lobe of abdominal segment X with 2 setae (*versus* 3 setae).

Moreover, detailed descriptions of immature stages of *Larinus* species are also important for further studies on generic and evidently also subgeneric taxonomic relationships within Lixini and to effectively protect endangered species and promote the use of larvae of *Larinus* species as potential biological control agents against weeds (e.g., *Carduus*, *Cirsium*, *Echinops*). Species identification of larvae with morphological evidence is relatively easy, and it is generally much cheaper than identification by molecular methods (Hirsch et al. 2010). The largest problem in the identification of the immature stages is the relatively low number of available larval descriptions in comparison to the many species only known at the adult stage. However, the problem is not exclusive to Curculionidae, being common to many other beetle groups.

**Table 1.** Larval setal index of species from the subgenus Larinus: (a) *L. capsulatus*, (b) *L. fucatus*, (c) *L. idoneus*, (d) *L. inaequalicollis*, (e) *L. latus*, (f) *L. pollinis*, (g) *L. sibiricus* and (h) *L. vulpes* (italics – micro-setae; Σ – uncountable, defined as more than 50; ? – no data).

Part of body		a	b	c	d	e	f	g	h
**Prothorax**	Pronotal	~24	~26	12	26–29	12	Σ	32	~30–35
	Pleural	18	2	2	11	2	5	2	20
	Eusternal	5	?	1	10	2	5	3	12
	Pedal area	12	?	7	11	8	12	7	10
**Meso- Metathorax**	Prodorsal	3–4	5–9	2	3	1	9	4	3
	Postdorsal	14	18	5	14	4	0	11–12	13
	Alar area	4	1	3	7	1	11	3–4	6–7
	Spiracular area	4–5	?	?	?	?		?	6
	Epipleural	4–5	5	1	8–9	4?	1–2	1	6–9
	Pleural	12	2	2	8	2	5	2	14
	Eusternal	5	6	1	4–5	2	5	2–3	13
	Pedal area	12	7	7	11–12	8	12	8	10
**Abd. segment I-VIII**	Prodorsal I-VII	3	6	2	3	1	9	5	2
	VIII	?	?	1	?	?	3	?	2
	Postdorsal I-VII	19	24	8	17	8	~40	17	13
	VIII	?	?	5	?	?	8	?	10
	Spiracular I-VII	3	3	2	6	1	9	3	7
	VIII	?	?	?	?	?	0	?	2
	Epipleural I-VII	8	3	2	12	2	6	3	10–14
	VIII	?	?	?	?	?	5	?	13
	Pleural I-VII	7	1	2	6	2	3	1	9
	VIII	?	?	?	?	?	3	?	9
	Laterosternal I-VII	1	1	?	1	0	2	0	2
	VIII	?	?	?	?	0	2	?	2
	Eusternal I-VII	4	*2*	*3*	2	0	3	2	2
	VIII	?	?	?	?	0	1	?	2
**Abd. segment IX**	Dorsal	5	?	6	12	5	6	7	7
	Lateral	9	?	2	9	2	1	2	13
	Sternal	11	?	2	3	2	1	1	4
**Abd. segment X**	Anal area	*4*	*4*	*1*	*2*	*4*	*3*	*2*	*3*
**Head capsule**	Dorsal	5	5	5	4	5	5	5	5
	Posterior	*2*	*2*	*0*	*1*	*0*	*0*	*1*	*3*
	Lateral	3	3	2	2	2	1	3	2
	Ventral	?	?	?	?	?	1	?	2
	Frontal	6	5	2	7	5	5	4	6
	Clypeal	2	2	2	2	2	2	2	2
	Labral	3	3	3	3	3	3	3	3
	Mandibular	2	2	2	0	2	2	0	2
**Epipharynx**	Anterolateral	2	3	4	4	4	5	3	4
	Anteromedial	?	2	2	3	4	2	2	3
	Medial	?	0	2	*2*	0	*2*	2	2
**Maxilla**	Lacinia (dorsal)	7	9	8	9	8	10	8	9
	Lacinia (ventral)	3	5	6	3	4	4	4	5
	Palpal	*1*	*1*	*1*	*1*	*1*	*1*	*1*	*1*
	Stipital	2	1	1	2	1	1	1	2
	Palpiferal	2	2	2	2	2	2	2	2
**Labium**	Postlabial	5	?	3	5	3	3	3	4
	Prelabial	1	1	1	1	1	1	1	1
	Ligular	*2*	*2*	*2*	*2*	*2*	*2*	*3*	*2*

**Table 2.** Pupal setal index of selected *Larinus* species: (a) *L. idoneus*, (b) *L. inaequalicolllis*, (c) *L. obtusus*, (d) *L. pollinis*, (e) *L. sibiricus*, (f) *L. sturnus*, (g) *L. turbinatus* and (h) *L. vulpes* (Σ – uncountable, defined as more than 50; ? – no data).

Part of body		a	b	c	d	e	f	g	h
**Head capsule**	vertical s.	-	1	1	1	2	1	-	1
	super orbital s.	1	2	2	3	2	2	1	2
	orbital s.	2	1	1	1	1	1	2	1
	post antennal s.	5	4	4	4	4	4	3	3
**Rostrum**	rostral s.	3	2	1	8	5	4	1	1
	epistomal s.	2	1	0	1	1	1	1	2
**Prothorax**	superapical s.	1	1	Σ	1	1	1	1	-
	apical s.	0	0	Σ	0	0	0	2	2
	lateral s.	~14	5	Σ	10	5	6	5	5
	superlateral s.	5	1	Σ	4	1	2	-	2
	discal s.	2	2	Σ	3	2	2	1	3
	posterolateral s.	19	4	Σ	21	4	4	3	6
**Mesothorax**		6	12	6	Σ	16	6	6	~14
**Metathorax**		6	12	6	Σ	16	6	6	~14
**Abd. segment I-VII**	dorsal	~20	9	Σ	~40	9	9	8	~25
	ventral	3	3	3	3	3	3	4	3
	lateral	~20	4	7	~20	4	4	2	3
**Abd. segment VIII**	dorsal	8	9	Σ	~10	8	8	8	~10
	ventral	3	3	Σ	3	3	3	4	4
	lateral	~10	4	7	~20	4	4	2	3
**Abd. segment IX**	ventral	?	2	3	5	4	4	?	?
	dorsal	?	1	2	Σ	1	3	?	?
**Legs-femoral**		3	3	3	6	3	3	3	3(4)

### Key to the immature stages of *Larinus*

**Larvae (last instar)**

The following key is based on the larvae of *Larinus vulpes* described in this paper and on 13 selected descriptions of larvae in the genus *Larinus* published previously (Lee and Morimoto 1988; Nikulina et al. 2004; Zotov 2009a, 2010; Gosik and Skuhrovec 2011; Nikulina and Gültekin 2014). Unfortunately, Lee and Morimoto (1988) only provide a general description of the genus for two species, and it was not possible to divide these two species (see aggregation of both species at point 12). Further comments are given in the previous section under Discussion.

**Table:** 

1	Body with high count of setae; pronotum with more than 25 setae, postdorsum on meso- and metathorax and also on abdominal segments I–VII with more than 12 setae	**2**
–	Body with usual count of setae; pronotum with less than 15 setae, postdorsum on meso- and metathorax and also on abdominal segments I–VII with fewer than 10(12) setae	**7**
2	Frons with 6 or 7 *fs.* Postlabium with 4 or 5 setae. Stipes with 2 long *sts.* Predorsum on meso- and metathorax with 3 *prs.* Dorsal part of body distinctly spiculate	**3**
–	Frons with 4 or 5 *fs.* Postlabium with 3 setae. Stipes with 1 long *sts.* Predorsum on meso- and metathorax up to 4 *prs.* Dorsal part of body not distinctly spiculate	**5**
3	Postepicranial setae *pes_1_–pes_2_* distinct. Frons with 6 *fs.* Endocarina not distinct, its length is as half-length of frons or less. Ligula with 2 very thin *ligs*	**4**
–	Postepicranial seta *pes_1_* very small, indistinct. Frons with 7 *fs.* Endocarina distinct, massive, approximately 2/3 the length of frons. Ligula with 1 micro *ligs* and two sensilla	**L. (L.) inaequalicollis**
4	Postlabium with 5 setae. Meso- and metathorax with 4 *as*, 4 *ss* and 5 *eps.* Abdominal segments I–VII with more than 15 *pds* and 8 or fewer *eps.* Lateral lobe of abdominal segment X with 3 setae	**L. (L.) capsulatus**
–	Postlabium with 4 setae. Meso- and metathorax with 6–7 *as*, 6 *ss* and 6–9 *eps.* Abdominal segments I–VII with fewer than 14 *pds* and more than 10 *eps.* Lateral lobe of abdominal segment X with 2 setae	**L. (L.) vulpes**
5	Frons with 5 setae. Endocarina either very thin and short (less than 1/3 of the length of frons) or absent. Prodorsum on metathorax with more than 5 *prs*	**6**
–	Frons with 4 setae. Endocarina distinct, length of approximately 1/3 of the length of frons. Prodorsum on metathorax with 4 *prs*	**L. (L.) sibiricus**
6	Endocarina very thin, length of less than 1/3 of the length of frons. Epipharynx with 3 *als.* Pronotum with 26 *prns*. Meso- and metathorax with 18 *pds*, and abdominal segments I–VII with approximately 24 *pds*	**L. (L.) fucatus**
–	Endocarina absent. Epipharynx with 5 *als.* Pronotum with more than 50 *prns*. Meso- and metathorax with more than 20 *pds*, and abdominal segments I–VII with approximately 40 *pds*	**L. (L.) pollinis**
7	Abdominal segments I–VII with more than 1 *prs*, 7 or 8 *pds*, and 1 or 2 *ss*	**8**
–	Abdominal segments I–VII without *prs*, only 5 *pds*, and 1 *ss* **L. (Larinomesius) obtusus**
8	Frons with 5 *fs.* Meso- and metathorax and also abdominal segments I–VII with 1 *prs.* Meso- and metathorax with 1 *as* and 2–3 *ss*	**9**
–	Frons with 2 *fs.* Meso- and metathorax and also abdominal segments I–VII with 2 *prs.* Meso- and metathorax with 3 *as* and 1 *ss*	**L. (L.) idoneus**
9	Meso- and metathorax with 5 *pds*, 1 *as* and 3 *ss.* Abdominal segments I–VII with 8 *pds.*	**10**
–	Meso- and metathorax with 4 *pds*, 1 *as* and 2–3 *ss.* Abdominal segments I–VII with 7 *pds* **11**
10	Head with wide, bright stripes on sides. Abdominal segments I–VII with 1 *eps.* Lateral lobes of abdominal segment X with 1 tiny seta	**L. (Phyllonomeus) sp. aff. leuzeae**
–	Head with barely noticeable bright stripes on sides. Abdominal segments I–VII with 2 *eps.* Lateral lobes of abdominal segment X with 4 setae	**L. (L.) latus**
11	Abdominal segments I–VII with 2 *eps* and 2 *ps* **12**
–	Abdominal segments I–VII with 1 *eps* and 1 *ps* **L. (Phyllonomeus) turbinatus**
12	Stipes with 1 long *sts.* Epipharynx with 4 *als.* Pronotum with 9 *prns*. Meso- and metathorax with 2 *ss* **L. (Phyllonomeus) meleagris, L. (Phyllonomeus) latissimus**
–	Stipes with 2 long *sts.* Epipharynx with 5 *als.* Pronotum with 11 *prns*. Meso- and metathorax with 3 *ss* **L. (Phyllonomeus) sturnus**

**Pupae**

The following key is based on the pupa of *L. vulpes* described in this paper and on seven descriptions of pupae of the genus *Larinus* published previously (Zotov 2009a, 2010; Gosik and Skuhrovec 2011).

**Table:** 

1	Pronotum with 25 or even more setae. *Sas* without special protuberances	**2**
–	Pronotum with fewer than 20 setae. *Sas* placed on thorn-like protuberances	**5**
2	Setae on pronotum form a regular pattern, which consists of lines and groups of setae (counting of setae possible)	**3**
–	Pronotum densely covered by very long setae (counting of setae impossible)	**L. (L.) pollinis**
3	Cuticle around setae not more pigmented than rest of pronotum. Rostrum with 3 or 4 *pas* and 3 or more *rs* **4**
–	Cuticle around setae dark-pigmented, visible spots formed. Rostrum with 3 *pas* and only 1 *rs* **L. (L.) sibiricus**
4	Head with 1 *sos*; *vs* absent. Rostrum with 5 *pas* and 3 *rs* **L. (L.) inaequalicolllis**
–	Head with 3 *sos*; *vs* present. Rostrum with 4 *pas* and 8 *rs* **L. (L.) vulpes**
5	Body rather elongated. Urogomphi distinct	**6**
–	Body rather stout. Urogomphi very short, almost not visible	**7**
6	Body length over 10 mm. *Sas* on head as horns. Urogomphi with dark, sclerotised apex	**L. (Phyllonomeus) sturnus**
–	Body length under 8 mm. *Sas* on head, no protuberance shaped as horns. Urogomphi without sclerotised apex	**L. (Phyllonomeus) turbinatus**
7	Head with 2 *os.* Rostrum with 3 *pas.* Pronotum with 3 *pls* **L. (L.) idoneus**
–	Head with 1 *os.* Rostrum with 4 *pas.* Pronotum with 4 *pls* **L. (Larinomesius) obtusus**

### Biology and ecology of *Larinus vulpes*

**Habitats.** *Larinus vulpes* occurred in the primary and degraded steppe lands, slopes, limestone and chalk cliffs of low mountains, forest edges, man-made treelines, roadsides and other ruderal plots. This weevil preferred open, sunny areas. In Iran, the weevil was recorded as high as 2580 m a. s. l. in the mountains (Gültekin and Podlussány 2012).

**Adult behaviour.** Adults feed on the upper surface of the leaf. As feeding was initiated, an adult raised and strongly lowered its head onto the leaf surface, which was followed by some motions of the mandibles and repeated “peck-like” motions by its rostrum. Apparently, the motion created additional pressure and helped to break through cuticle and epidermis covered with woolly hairs. Following this behaviour, an imago gnawed on mesophyll tissue, moving the head away from itself, and at one feeding, a weevil could gnaw out an irregularly shaped piece of leaf (approximately 2 × 8 mm). The translucent cuticle of the leaf downside covered with dense woolly hairs remained intact (Fig. 22). During feeding, some short pauses by the weevil were observed. After eating, the weevil cleaned the apex of its rostrum using the apexes of both tibias. Weevils moved from one flower head to another by walking; they flew very reluctantly.

**Host plant.** Both adults and larvae were recorded feeding exclusively on *Echinops ruthenicus* and *E. sphaerocephalus*. We never observed *L. vulpes* on other plant species. According to Zwölfer (1985) and Nicolas (1895), weevils feed on *Echinops microcephalus* Sibth. and Sm. and *E. ruthenicus* (as *E. ritro* L.). In this case, *L. vulpes* is oligophagous (or monophagous *sensu* Jolivet 1992). Imagines fed on the leaves and on the apexes of the stems, and larvae gnawed tissues in the flower head. Hoffmann (1954) noted that *L. vulpes* were often recorded on *Cirsium ferox*, but whether this plant was a host for the weevil was not resolved.

**Figures 21–28.**
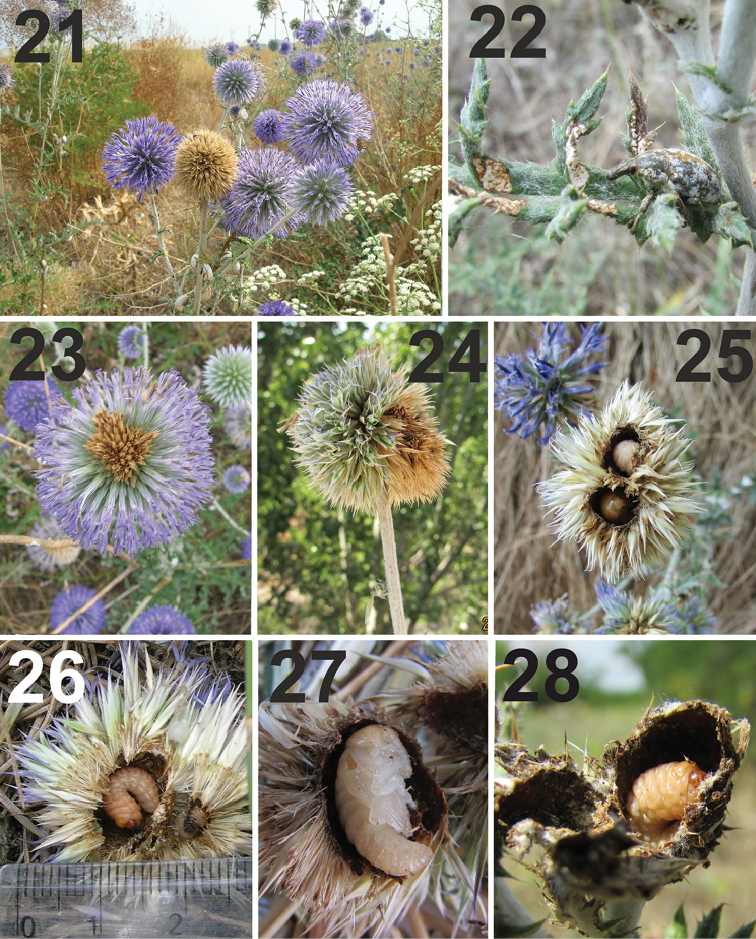
Habitat, life cycle, and immature stages of *Larinus vulpes*. **21** Habitat with flowering *Echinops ruthenicus* (Cyrilivska Spit) **22** Adult and the damaged leaf of *Echinops ruthenicus* by weevil **23** When larva begins its development, upper parts of the flower head die and are visible as a light brown spot **24** Pupa cell results in deforming of flower head **25** Larva and pupa in the same inflorescence **26** Two larvae in the same flower head, right larva is younger and dead **27** Pupa in the inflorescence **28** Mature larva in a chamber outside a flower head. All photos SV Volovnik.

**Life cycle.** In Ukraine, adults were recorded from the end of April (usually from the end of May) onwards. The primary peak in the population of adults was reached at the end of June and then decreased. Active imagines from the new generation were observed from the beginning of August to early September. The phenology of the weevil is closely synchronized with the phenology of its host plant (Volovnik 1996). In spring, the weevils began to feed first on the rosettes of host plants and then gnawed the young stems and leaves (Fig. 22). *Larinus vulpes* is a univoltine species. Mating and oviposition occurred from the second part of June to the middle of July. The common scenario of oviposition and subsequent development from egg to adult were the typical for the genus *Larinus* (Volovnik 2016).

Female *L. vulpes* preferred to lay eggs in the larger flower heads on the side stems (Volovnik 2016). In this case, the female makes a hole in the flower head. Freshly laid, eggs were oval, milky white, glossy, 1.3–1.6 mm long and 0.7–1.2 mm wide. The eggs were laid solitarily in the deepening in the receptacle, gnawed by the female, or very close to the receptacle (maybe, the rostrum of some weevils was too short for access). The process of oviposition has been described in detail previously (Volovnik 1996). Eggs were found in inflorescences from the end of June to the end of July. Very occasionally, dry fragments of the egg and also a small larva were found in the same flower head. In other instances, the fragments of eggs were recorded in the inflorescence but without any traces of weevil larvae. These eggs were likely destroyed by parasites or abiotic factors (i.e., desiccation).

**Figures 29–37.**
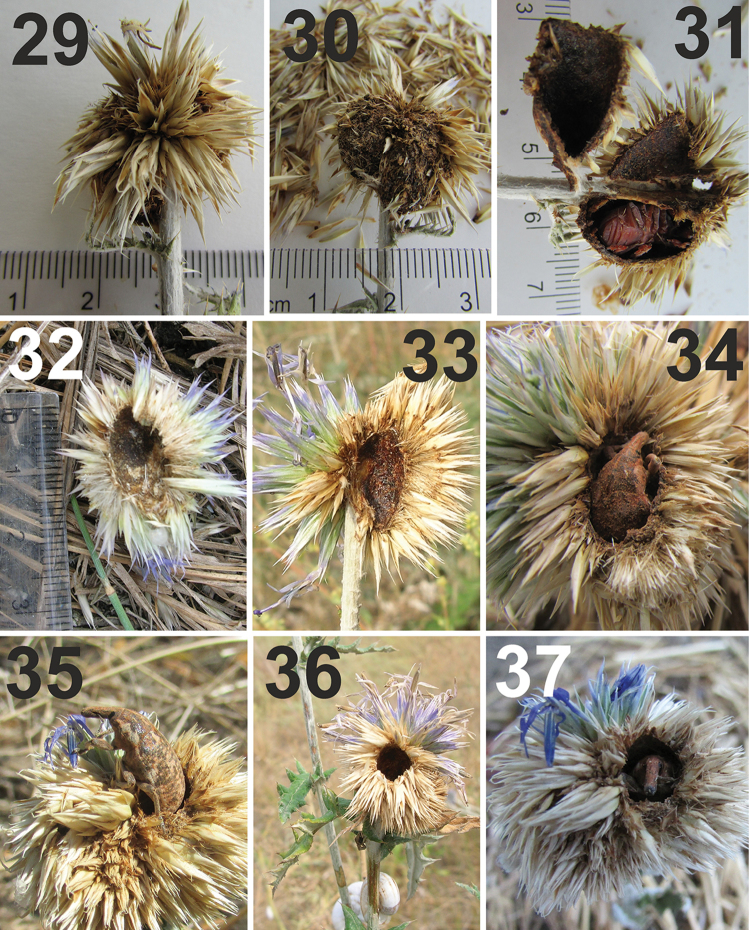
Hatching, pupal cells, and adults of *Larinus vulpes*. **29** Dead inflorescence with pupal cell inside **30** Pupation cell and dry fragments removed from outside of the cell **31** Pupation cell with a fresh, not fully coloured adult **32, 33** Pupation cell at the beginning (left) and after finishing construction. The inner layer of the finished wall is hard and glanced **34** Adult in pupation cell **35** Adult leaving the pupa cell **36** Exit hole of adult of new generation **37** Fresh adult in pupa cell. All photos: SV Volovnik.

Later, after hatching of larva, the oviposition site became a visible, brownish tiny spot (Fig. 23). Apparently, this spot was a result of damage to the flower head by the larva. Larvae nibbled the receptacle of the flower head and the bases of primary capitula, with the damaged capitula fixed with a sticky liquid that flowed out of receptacle, resulting in a deformed flower head (Fig. 24). The mature larva colouration was peach-orange (Figs 26, 28) and that of the young pupa yellow-orange (Figs 25, 27). Fabre (1922) wrote that he found “six and more” larvae in the same inflorescence and that three larvae in the same flower head “frequently happens”. Our data differed greatly from the observations of Fabre (see Table 3). Apparently, two larvae could finish full development in a medium-sized flower head (Fig. 26). Larvae were recorded from the end of June to the end of August. Larval development has been described and discussed in detail by Fabre (1922). According to direct observations by Fabre, larvae feed primarily on the sap of the receptacle. Mature larvae build a pupation cell (4–5 × 7–10 mm) (Figs 29–31), with the walls significantly stronger than those of the larval cell (Figs 32–33).

After emergence, adults remained in the dry inflorescence for 5–7 days until fully sclerotised (Fig. 37), with an upper surface that was usually densely covered with a rust, pollen-like flush (Figs 34–36). Adults hibernated outside host plants, most likely in the top layer of soil or among dry plant debris.

**Table 3.** Abundance of *Larinus vulpes* larvae in flower heads of *Echinops sphaerocephalus* (Khomutovskyi Step Reserve, 30–31.07.1982; n=77).

Count of larvae in one flower head	0	1	2	3	4	5
Count of the inflorescences with specific count of larvae	13	58	1	4	–	1

**Figures 38–40.**
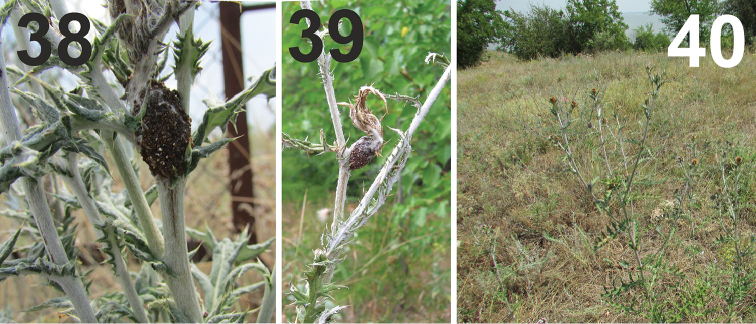
Pupal chamber outside inflorescence and habitat in 2006. **38, 39** Pupal chamber in the axil of a leaf **40** Habitat in dry, hot season in 2016; flower heads were small and died early. All photos SV Volovnik.

**Preimaginal development outside of flower heads.** In the second part of the summer in 2015, the second author found seven chambers on the side of the stem of *Echinops ruthenicus*. All were located 1–2 cm below the inflorescence. Two of the seven capsules were located on the same stem and touched one another. The largest chamber was approximately 0.7 × 1.25 cm. The walls of all chambers were solid but rather brittle and easily crushed by the fingers. The material of the walls had no taste. The external surface of the capsule wall was brown to black, rough, earthy coloured, lumpy and mat; with incrustations of elements characteristic for *Echinops*, i.e., wool-like coating of the stems, small leaves with spines and tips of spines (Figs 28, 38–39).

The inner surface of the wall was denser than that of the external item and was bright red-brown with yellowish spots and veins, glossy, and 0.8–1.0 mm thick. The inner surface was also smooth and appeared varnished and polished, similar to inner surface of the wall of the usual pupa chamber of *L. vulpes*, but without any incrustations. The contents of the chambers included the following: 2 chambers with dead imagines, 1 chamber with a living imago, one with only the head capsule of a larva, one chamber with a mature larva alive (Fig. 34), one with only pieces of a pupa and one with only parts of exuviae or cuticle remains. No excrement was observed but clearly the larvae incorporated their faeces into the wall during construction.

**Biotic interactions.** A parasitic wasp, *Bracon urinator* (Fabricius, 1898) (Hymenoptera: Braconidae), was reared from the pupae of *L. vulpes*. Sometimes the capitulum dried out, cracking the walls of larval or pupal cells, and ants, *Formica imitans* Ruzsky, 1902, destroyed the larvae and pupae of the weevil. Feeding on the inflorescences of *Echinops*, the rose beetle, *Protaetia metallica* (Herbst, 1782) (Coleoptera: Scarabaeidae), harmed larvae of *L. vulpes* developing in the same inflorescence. We found cells with dead weevil larvae together and simultaneously with living larvae or adults of carnivorous bugs, *Orius* sp. (Heteroptera: Anthocoridae) (Volovnik 1994).

***Larinus vulpes* and its host-plants**. Larvae of *L. vulpes* living in flower heads of globe thistle consumed the ovaries and unripe seeds. The prevalence of weevils in globe thistles sometimes reached 33% of inflorescences (SV, unpublished data). Some flower heads lost all their seeds, although in an overall view, the loss of some seeds may be expected. Globe thistles have a special morphological structure that separates the compound flower head into small, single primary capitula with one seed each (Mulkijanian 1951). The durable walls of the pupal cell were very strong, which might prevent the possible separation of each capitulum. Furthermore, Mulkijanian (1951) also recorded stems breaking in the wind, primarily when larvae damaged the receptacle. Most likely, the larvae of *Larinus vulpes* or some similar species of *Larinus* damaged the receptacles. Clearly, the specialisation of globe thistles to a specific environment, such as rocky slopes, also constrained the weevils, being adapted to that same environment. The chemical composition of *Echinops* is also somewhat specific, and some species in this genus have insecticidal, nematicidal, antifungal, bactericidal and also antiviral properities (i.e. Fokialakis et al. 2006; Zhanget al. 2009; Abdelnabby and Abdelrahman 2012; Tekwu et al. 2012; Gemechu et al. 2013; Liu et al. 2013). Therefore, the inhabitants of *Echinops* flower heads might also be protected from some parasites and predators because of the chemical compounds in the plant. This aspect requires further experimental investigations.

The absence of preimaginal development outside of flower heads is a characteristic of not only *L. vulpes* but also for the genus *Larinus* in general. Only six *Larinus* species construct analogous capsules (pupal chambers) with a sweet taste (known as “trehala”) on plant stems, namely: *L. capsulatus* Gültekin, 2008; *L. hefenborgi* Boheman, 1845; *L. nidificans* Guibourt, 1858; *L. ruficollis* Petri, 1907 (Gültekin 2008); *L. trehalanus* Gültekin and Shahreyary-Nejad, 2015 (Gültekin and Shahreyary-Nejad 2015); and *L. onopordi* (Fabricius, 1787) (Khnzoryan 1951). According to Zwölfer et al. (1971), larvae of *L. vulpes* sometimes construct capsules on the stems, but the authors did not provide further details for this observation. Fabre (1922) once recorded the larval chamber built in the axil of the leaf of *Echinops* sp. Of note, *L. vulpes* and the trehala building species develop on plants of the same genus, *Echinops*, and perhaps the construction activity outside of flower heads might be to avoid properties of the thick adhesive sap of these plants. Of the seven *Larinus* species that develop on *Echinops* spp., all construct outer pupal chambers (*L. vulpes* included). According to Iljin (1953), *E. ruthenicus* and other globe thistles contain the type of rubber that turns into a dense substance when exposed to air. Closely related to *Larinus* weevils, *Afrolarinus moestus* (Chevrolat, 1882) also develops on *Echinops*, but only in the flower heads (Gültekin 2013).

Two important moments in the life cycle of *Larinus vulpes* remain unclear: (1) why do eggs appear outside flower heads? (2) can imagines open their chambers on the stems and emerge at the proper time? According to the assumptions of Fabre (1922), an egg could fall from the inflorescence after being laid, or the female could lay an egg in an unusual place “either by inadvertence or by intention”. This event is unlikely because females preferred to lay eggs in the top of flower heads. Occasionally, females laid an egg on a lateral part but never on the bottom of flower head. Thus, falling out into the axil of a leaf is doubtful. Unlikely also was “inadvertence”, because this phenomenon is not that rare. On a relatively small patch of grassland (approximately 150 m²), seven chambers were found. Therefore, females laid eggs in an unusual place, for enigmatic reasons. In previous years, Volovnik conducted direct observations of *L. vulpes* in the field but never saw its capsules on plant stems. In the region of investigation, the growing season for vegetation during 2016 was abnormally dry and hot. Most of the globe thistles were short with small inflorescences and died earlier than usual (Fig. 40). It is possible that such an extreme situation resulted in an acute shortage of suitable places for normal oviposition; thus, a significant portion of eggs were laid in unconventional places.

## Supplementary Material

XML Treatment for Larinus (Larinus) vulpes

## References

[B1] Abdelnabby HM Abdelrahman SM (2012) Nematicidal activity of selected flora of Egypt. Egyptian Journal of Agronematology 11: 106–124.

[B2] Chopik VI Dudchenko LG Krasnova AN (1983) Wild useful plants of Ukraine. Guide. Kyiv, Naukova Dumka Publishing, 399 pp. [In Russian]

[B3] Csiki E (1934) Coleopterorum Catalogus auspiciis et auxilio W. Junk editus a S. Schenkling. Pars 134. Curculionidae: subfam. Cleoninae. Junk, Berlin, 152 pp.

[B4] Czarapata EJ (2005) Invasive Plants of the Upper Midwest. An Illustrated Guide to Their Identification and Control. Madison, University of Wisconsin Press, 236 pp.

[B5] Dedyukhin SV (2011) Pecularities of fauna of the phytophagous beetles (Coleoptera, Chrysomeloidea) in the north region of Kungursky island forest-steppe. Bulletin of Moscow Society of Naturalists, Biological series 116: 20–28. [In Russian]

[B6] Eram S Ahmad M Arshad S (2013) Experimental evaluation of *Echinops echinatus* as an effective hepatoprotective. Scientific Research and Essays 8: 1919–1923.

[B7] Fabre JH (1922) The life of the weevil. New York, Dodd, Mead and Co., 346 pp https://doi.org/10.5962/bhl.title.56720

[B8] Fokialakis N Cantrell ChL Duke SO Skaltsounis AL Wefge DE (2006) Antifungal Activity of *Thiophenes* from *Echinops ritro*. Journal of Agricultural and Food Chemistry 54: 1651–1655. https://doi.org/10.1021/jf052702j 16506815 10.1021/jf052702j

[B9] Gardner JCM (1934) Immature stages of Indian Coleoptera (14) (Curculionidae). Indian Forest Records 20(2): 1–48.

[B10] Gemechu F Sori W Santiago DR (2013) Efficacy of botanical powders and cooking oils against Angoumois grain moth, *Sitotroga cereallela* O. (Lepidoptera: Gelechiidae) in stored maize. African Journal of Biotechnology 12: 1978–1986. https://doi.org/10.5897/AJB12.2548

[B11] Gosik R Skuhrovec J (2011) Descriptions of mature larvae and pupae of the genus *Larinus* (Coleoptera: Curculionidae, Lixinae). Zootaxa 3019: 1–25.

[B12] Gosik R Wanat M (2014) Descriptions of immature stages of the weevil *Lixus punctiventris* Boheman, 1835 (Coleoptera, Curculionidae, Lixini). Zootaxa 3754(2): 159–172. https://doi.org/10.11646/zootaxa.3754.2.5 24869687 10.11646/zootaxa.3754.2.5

[B13] Gültekin L (2006) A new species of the weevil genus *Larinus* Dejean from Turkey and Syria (Coleoptera: Curculionidae: Lixinae). Zootaxa 1248: 21–26.

[B14] Gültekin L (2008) Taxonomic review of the stem-inhabiting trehala-constructing *Larinus* Dejean, 1821 (Coleoptera: Curculionidae): New species, systematics and ecology. Zootaxa 1714: 1–18.

[B15] Gültekin L (2013) *Afrolarinus*, a new genus of Lixini (Coleoptera: Curculionidae: Lixinae) from Afrotropical region with taxonomic revision. Deutsche Entomologische Zeitschrift 60: 251–260.

[B16] Gültekin L Fremuth J (2013) Lixini In: Löbl I, Smetana A (Eds) Catalogue of Palaearctic Coleoptera, Volume 8, Curculionoidea II. Brill, Leiden, pp 456–471.

[B17] Gültekin L Podlussány A (2012) New Faunistic Data on Selected Palaearctic Species of the Genus *Larinus* Dejean, 1821 (Coleoptera: Curculionidae, Lixinae). Journal of the Entomological Research Society 14: 71–85.

[B18] Gültekin L Shahreyary-Nejad S (2015) A new trehala-constructing *Larinus* Dejean (Coleoptera: Curculionidae) from Iran. Zoology in the Middle East 61: 246–251. https://doi.org/10.1080/09397140.2015.1069240

[B19] Hirsch J Sprick P Reineke A (2010) Journal of Economic Entomology 103: 898–907. https://doi.org/10.1603/EC09381 20568637 10.1603/ec09381

[B20] Hoffmann A (1954) Coléoptères Curculionides. (Deuxième Partie). Faune de France 59: 1–1208.

[B21] Horn G Kupfer A Kalbitz J Gerdelbracht H-J Kluge H Eder K Dräger B (2008) Great globe thistle fruit (*Echinops sphaerocephalus* L.), a potential new oil crop. European Journal of Lipid Science and Technology 110: 662–667. https://doi.org/10.1002/ejlt.200700142

[B22] Hymete A Rohloff J Kjøsen H Iverson T (2005) Acetylenic thiophenes from the roots of *Echinops ellenbeckii* from Ethiopia. Natural Product Research 19: 755–761. https://doi.org/10.1080/1478641042000301711 16317830 10.1080/1478641042000301711

[B23] Iljin MM (1953) Rubber plants of the flora of the USSR. In: Rubber and Rubber Plants. Volume 2. Academy of Science of the USSR Publishing Company, Moscow–Leningrad, 9–104. [In Russian]

[B24] Jabłoński B Kołtowski Z (2005) Nectar secretion and honey potential of honey plants growing under Poland’s conditions. Journal of Apicultural Science 49: 59–63.

[B25] Jolivet P (1992) Insects and plants: parallel evolution and adaptations, 2^nd^ ed. Sandhill Crane Press, Inc. , Gainesville, Florida, 190 pp.

[B26] Kadereit JW Jeffrey Ch (Eds) (2007) Flowering Plants. Eudicots: Asterales. Springer–Verlag, Berlin, Heidelberg, 636 pp.

[B27] Karunamoorthi K Hailu T (2014) Insect repellent plants traditional usage practices in the Ethiopian malaria epidemic-prone setting: an ethnobotanical survey. Journal of Ethnobiology and Ethnomedicine 10: 22. https://doi.org/10.1186/1746-4269-10-22 10.1186/1746-4269-10-22 PMC3932844 24521138

[B28] Khnzoryan SM (1951) Notes of weevil fauna of Armenia (Coleoptera, Curculionidae). Izvestia Akademii nauk Armyanskoj SSR, biologicheskie i selskokhozyajstvennie nauki 4: 827–831. [In Russian]

[B29] Kolomiychuk V Vynokurov D (2016) Syntaxonomy of the *Festuco*-*Brometea* class vegetation of the Azov sea coastal zone. Haquetia 15: 79–104.

[B30] Lee CY Morimoto K (1988) Larvae of the family Curculionidae of Japan. Part 2. Hyperinae to Cioninae (Insecta: Coleoptera). Journal of the Faculty of Agriculture Kyushu University 33(1–2): 131–152.

[B31] Liu X Hao X Zhou L Zhi L (2013) GC-MS Analysis of Insecticidal Essential Oil of Aerial Parts of *Echinops latifolius* Tausch. Journal of Chemistry 249182: 1–6.

[B32] Marvaldi AE (1998) Larvae of Entiminae (Coleoptera: Curculionidae): Tribal diagnoses and phylogenetic key, with a proposal about natural groups within Entimini. Entomologica Scandinavica 29: 89–98. https://doi.org/10.1163/187631298x00212

[B33] Marvaldi AE (1999) Morfología larval en Curculionidae (Insecta: Coleoptera). Acta zoológica Lilloana 45(1): 7–24.

[B34] May BM (1977) Immature stages of Curculionidae: larvae of soil dwelling weevils of New Zealand. Journal of the Royal Society of New Zealand 72: 189–228. doi: 10.1080/03036758.1977.10427160

[B35] May BM (1993) Fauna of New Zealand, 28. Larvae of Curculionoidea (Insecta: Coleoptera): a systematic overview. Manaaki Whenua Press, Lincoln, New Zealand, 226 pp.

[B36] May BM (1994) An introduction to the immature stages of Australian Curculionoidea. In: Zimmerman EC (Ed.) Australian weevils (Coleoptera: Curculionidae). Vol. 2. CSIRO, Melbourne, 533–535.

[B37] McClay AS (1988) The potential of *Larinus planus* (Coleoptera: Curculionidae), an accidentally introduced insect in North America, for biological control of *Cirsium arvense* (Compositae). Proceedings of the VII International Symposium on Biological Control of Weeds. E.S. Delfosse (Ed.) in Instituto Sperimentale per la Patologia Vegetale Ministero dell’ Agricoltura edelle Foreste 1990, 173–179.

[B38] Mulkijanian YaI (1951) Biology of reproduction and phenology of the globe thistles (*Echinops* L.). Bulletin of botanical garden of Academy of Sciences of Armenian SSR 12: 79–87. [In Russian]

[B39] Murch S Wierenga E El-Demerdash M Saxena PK (2003) *In vitro* propagation of the Egyptian medicinal plant, *Echinops spinosissimus* Turra. Plant Cell, Tissue and Organ Culture 74: 81. https://doi.org/10.1023/A:1023398606293

[B40] Nicolas H (1895) Larves et nymphes de certains *Larinus* se développant sur les chardons (Cynarocephale) de nos régions (Avignon). Miscellanea Entomologica 3(8): 89–91.

[B41] Nikulina ON (2001) Larval morphology of the weevil genus *Lixus* (Coleoptera, Curculionidae) from Middle Asia. Entomological Review 81: 809–823. [Original text published in Russian in Zoologicheskiy Zhurnal 80 (10): 183–195]

[B42] Nikulina ON (2007) New data on larvae of weevils of the genus *Lixus* (Coleoptera, Curculionidae) from Central Asia. Entomological Review 87: 750–756. [Original text published in Russian in Zoologicheskiy Zhurnal 86(9): 1086–1092] https://doi.org/10.1134/s0013873807060103

[B43] Nikulina ON Gültekin L (2011) Larval morphology of *Lixus cardui* Olivier and *Lixus filiformis* (Fabricius) (Coleoptera: Curculionidae): biological control agents for Scotch and musk thistles. Australian Journal of Entomology 50: 253–257. https://doi.org/10.1111/j.1440-6055.2011.00810.x

[B44] Nikulina ON Gültekin L (2014) New data on the Larvae of the Weevil Genus *Larinus* Dejean, 1821 (Coleoptera, Curculionidae) from Northeastern Turkey. Entomological Review 94: 1010–1018. [Original text published in Russian in Zoologicheskii Zhurnal 93(5): 641–650] https://doi.org/10.1134/S0013873814070100

[B45] Nikulina O Gültekin L Güçlü S (2004) Larval morphology of the capitulum weevil, *Larinus latus* (Herbst) (Coleoptera, Curculionidae). New Zealand Journal of Zoology 31: 23–26. https://doi.org/10.1080/03014223.2004.9518355

[B46] Parhat R Makabel B Nurhabek U Tohonrbek A Hayni Song FF Baysanbek A Yang HY Ding G Zou ZM (2014) Overview of application and research of *Echinops* genus in Chinese medicine. Zhongguo Zhong yao za zhi = China Journal of Chinese Materia Medica 39: 3865–3869. 25612456

[B47] Reddy CS Bagagyanarayana G Reddy KN Raju VS (2008) Invasive alien flora of India. National Biological Information Infrastructure, US Biological Survey, USA, 43 pp.

[B48] Scherf H (1964) Die Entwicklungsstadien der mitteleuropäischen Curculioniden (Morphologie, Bionomie, Ökologie). Abhandlungen der Senckenbergischen Naturforschenden Gesellschaft 506: 1–335.

[B49] Seastedt TR Knochel DG Garmoe M Shosky SA (2007) Interactions and effects of multiple biological control insects on diffuse and spotted knapweed in the Front Range of Colorado. Biological Control 42: 345–354. https://doi.org/10.1016/j.biocontrol.2007.06.003

[B50] Skuhrovec J Gosik R Caldara R (2014) Immatures of Palaearctic species of the weevil genus *Tychius* (Coleoptera, Curculionidae): new descriptions and new bionomic data with an evaluation of their value in a phylogenetic reconstruction of the genus. Zootaxa 3839(1): 1–83. https://doi.org/10.11646/zootaxa.3839.1.1 25081903 10.11646/zootaxa.3839.1.1

[B51] Skuhrovec J Gosik R Caldara R Košťál M (2015) Immatures of Palaearctic species of the weevil genus *Sibinia* (Coleoptera, Curculionidae): new descriptions and new bionomic data with suggestions on their potential value in a phylogenetic reconstruction of the genus. Zootaxa 3955(2): 151–187. https://doi.org/10.11646/zootaxa.3955.2.1 25947846 10.11646/zootaxa.3955.2.1

[B52] Skuhrovec J Volovnik S (2015) Biology and morphology of immature stages of *Lixus canescens* (Coleoptera: Curculionidae: Lixinae). Zootaxa 4033(3): 350–362. https://doi.org/10.11646/zootaxa.4033.3.2 26624410 10.11646/zootaxa.4033.3.2

[B53] Stejskal R Trnka F Skuhrovec J (2014) Biology and morphology of immature stages of *Coniocleonus nigrosuturatus* (Coleoptera: Curculionidae: Lixinae). Acta Entomologica Musei Nationalis Pragae 54(1): 337–354.

[B54] Stève D Blandine MKL Michel L Clergé Tch (2016) Variability in the spice compositions of *nkwi* in Western Cameroon: Difficulties in local formulations. International Journal of Food Science and Nutrition 1: 21–29.

[B55] Tekwu EM Askun T Kuete V Nkengfack AE Nyasse B Etoa FX Beng VP (2012) Antibacterial activity of selected Cameroonian dietary spices ethno-medically used against strains of *Mycobacterium tuberculosis*. Journal of Ethnopharmacology 142: 374–382. https://doi.org/10.1016/j.jep.2012.05.003 22595661 10.1016/j.jep.2012.05.003

[B56] Ter-Minassian ME (1967) Zhuki-dolgonosiki podsemejstva Cleoninae fauny SSSR. Tsvetozhily i stebleedy (triba Lixini). Nauka, Leningrad, 140 + 1 p. [English translation: *Weevils of the Subfamily Cleoninae in the Fauna of the USSR. Tribe Lixini*. 1978USDA Agricultural Research Service, Washington, D. C. by Amerind Publishing Co. Pvt. Ltd., New Delhi,. 166 pp]

[B57] The Green Vision Newspaper (2017) How *Echinops* is bringing in new revenue through ABS. https// www.greenvision.news/how-echinops-is-bringing-in-new-revenue-through-abs/[accessed 11.II.2017]

[B58] Trnka F Stejskal R Skuhrovec J (2015) Biology and morphology of immature stages of *Adosomus roridus* (Coleoptera: Curculionidae: Lixinae). Zootaxa 4021(3): 433–446. https://doi.org/10.11646/zootaxa.4021.3.3 26624140 10.11646/zootaxa.4021.3.3

[B59] Trnka F Stejskal R Skuhrovec J (2016) The morphology of the immature stages of two rare *Lixus* species (Coleoptera, Curculionidae, Lixinae) and notes on their biology. ZooKeys 604: 87–116. https://doi.org/10.3897/zookeys.604.9018 10.3897/zookeys.604.9018 PMC4978021 27551208

[B60] Volovnik SV (1994) On parasites and predators of Cleoninae weevils (Col. Curculionidae) in Ukrainian steppe. Anzeiger für Schädlingskunde. , Pflanzenschutz, Umweltschutz 67: 77–79. https://doi.org/10.1007/BF02020366

[B61] Volovnik SV (1996) Distribution and ecology of some species of Cleoninae (Coleoptera, Curculionidae) III. Genus *Larinus* Germ. Entomological Review 75: 10–19. [Original Russian text published in Entomologicheskoe Obozrenie, 1995, 74: 314–321]

[B62] Volovnik SV (2016) On Oviposition in Weevils of the Genus *Larinus* Dej. (Coleoptera, Curculionidae): Entomological Review 96: 309–317. [Original Russian text published in Entomologicheskoe Obozrenie, 2016, 95: 58–70] https://doi.org/10.1134/S0013873816030088

[B63] Wiersema JH León B (2016) World Economic Plants: A Standard Reference, 2^nd^ ed. CRC Press, Boca Raton, Florida, 336 pp.

[B64] Woodburn TL Briese DT (1996) The contribution of biological control to the management of thistles. Plant Protection 11: 250–253.

[B65] Wroblewska A Ayers GS Hoopingarner RA (1993) Nectar production dynamics and bee reward – a comparison between Chapman honey plant (*Echinops spaerocephalus* L.) and blue globe thistle (*Echinops ritro* L.). American Bee Journal 133: 789–796.

[B66] Zhang P Liang D Jin W Qu H Cheng Y Li X Ma Zh (2009) Cytotoxic *Thiophenes* from the Root of *Echinops grijisii* Hance. Zeitschrift für Naturforschung 64c: 193–196. https://doi.org/10.1515/znc-2009-3-407 10.1515/znc-2009-3-407 19526711

[B67] Zotov AA (2009a) Morphology of the preimaginal stages of three species of weevil of the *Lixini* (Coleoptera: Curculionidae). Kavkazskiy Entomologicheskiy Byulleten – Caucasian Entomological Bulletin 5(1): 81–90. [In Russian]

[B68] Zotov AA (2009b) Morphology of the preimaginal stages of weevil *Lixus iridis* Olivier, 1807 (Coleoptera: Curculionidae). Kavkazskiy Entomologicheskiy Byulleten – Caucasian Entomological Bulletin 5(2): 249–252. [In Russian]

[B69] Zotov AA (2010) Morphology of preimaginal stages of the genus *Larinus* Dejean, 1821 (Coleoptera: Curculionidae). Part I. Kavkazskiy Entomologicheskiy Byulleten – Caucasian Entomological Bulletin 6(2): 171–178. [In Russian]

[B70] Zotov AA (2011) Morphology of the preimaginal stages of weevils of the tribe Cleonini *sensu lato* (Coleoptera: Curculionidae). Kavkazskiy Entomologicheskiy Byulleten – Caucasian Entomological Bulletin 7(2): 153–162. [In Russian]

[B71] Zwölfer H (1988) Präadaptation, Wirtskreiserweiterung and Parallel-Cladogenese in der Evolution von phytophagen Insekten. Journal of Zoological Systematics and Evolutionary Research 26: 320–340. https://doi.org/10.1111/j.1439-0469.1988.tb00321.x

[B72] Zwölfer H Frick KE Andres LA (1971) A study of the host plants relationships of European members of the genus *Larinus* (Col: Curculionidae). Technical Bulletin of the Commonwealth Institute for Biological Control 14: 97–143.

